# Contextual factors associated with walking performance after stroke: a systematic review and meta-analysis

**DOI:** 10.3389/fneur.2025.1635024

**Published:** 2025-09-24

**Authors:** Yi Zhang, Lan Xu, Xi Pan, Lei Chen, Weiying Zhong, Jiaxuan Li

**Affiliations:** The First Affiliated Hospital of Soochow University, Suzhou, China

**Keywords:** stroke, walking performance, contextual factor, meta-analysis, systematic review

## Abstract

**Objective:**

To describe the walking performance of patients with stroke and summarize the contextual factors associated with walking performance.

**Methods:**

PubMed, Web of Science, Embase, and the Cochrane Library were searched from the inception to September 4, 2024. Observational studies on the walking performance of people with stroke and their contextual factors were eligible for inclusion. The relationship between contextual factors and walking performance was evaluated using Fisher’s *z*-value, which was then converted to correlation values (*r*).

**Results:**

Thirty studies were included. Walking performance measures included step count, time, distance, and bouts; step count was most common, with pooled results showing a mean 4,296 steps/day after stroke. Guided by the contextual factors classification framework, we stratified the contextual factors of walking performance among subjects with stroke into three dimensions: user context, environmental context, and task context. Meta-analysis showed that walking endurance (*r* = 0.60; 95% CI, 0.46 to 0.71) was strongly correlated with the number of daily steps. Moderate correlations were found between daily step counts and gait speed (*r* = 0.41; 95% CI, 0.23 to 0.56), quality of life (*r* = 0.46; 95% CI, 0.34 to 0.56), self-efficacy (*r* = 0.30; 95% CI, 0.21 to 0.39), cardiorespiratory fitness (*r* = 0.36; 95% CI, 0.09 to 0.59), balance (*r* = 0.37; 95% CI, 0.02 to 0.65), and Rivermead Motor Assessment (*r* = 0.49; 95% CI, 0.26 to 0.65). Furthermore, age (*r* = −0.10; 95% CI, −0.18 to −0.02) and area deprivation index (*r* = −0.15; 95% CI, −0.24 to −0.06) were associated with the number of daily steps after stroke, but effect sizes were small.

**Conclusion:**

Daily step counts among individuals with stroke were not guideline-compliant. Contextual factors can inform the design of context-aware interventions aimed at increasing daily step counts.

## 1 Introduction

Stroke is the second leading cause of death and the third leading cause of disability worldwide (1). Physical activity (PA) is defined as physical movement in which skeletal muscle contraction induces energy expenditure (2). PA has a robust health-promoting effect and improves the prognosis of patients with stroke. Adequate PA can improve cardiovascular health (3) and promote the functional recovery of patients with stroke (4, 5). In addition, PA can decrease negative emotions (depression, anxiety, etc.) after stroke (6, 7). Walking is the most frequent form of physical activity among individuals with stroke. In contrast to walking capacity (capability of a walking task in a clinical setting), walking performance refers to walking activities (i.e., daily steps, walking distance or duration of walking) in real-world settings (8). However, existing studies suggest that the walking performance of patients with stroke is much less than that recommended by the guidelines (9, 10). Therefore, improving walking performance among patients with stroke remains a priority for rehabilitation after stroke. Recently, mobile health (mHealth) has gradually become a research hotspot in the field of walking performance intervention because it fits the spatial and temporal dynamic characteristics of walking activities. Context-aware intervention is an emerging mobile intervention design that dynamically adjusts interventions based on an individual’s contextual information. Its core lies in the real-time optimization of intervention timing or content according to an individual’s contextual factors, which are defined as all information describing the status of entities, to enhance the accuracy, personalization, and effectiveness of the intervention (11, 12). Understanding and accurately obtaining contextual information can optimize intervention decisions. A systematic review indicated that context-aware interventions can effectively support behavioral changes to improve the health behaviors of users (13). However, the key contextual determinants of walking performance after stroke currently remain unclear.

To date, systematic reviews in this field have predominantly focused on physical activity (PA) among post-stroke individuals and its contextual factors, with no emphasis on walking performance as a distinct outcome. For instance, a systematic review by Thilarajah et al. (14) reported that factors such as age, gender, physical function, depression, fatigue, self-efficacy, and quality of life were significantly associated with PA. Although a number of studies included in this review utilized walking performance as an indicator of PA, their conclusions cannot be directly extrapolated to walking performance. This constraint, specifically, stems from the absence of subgroup analyses stratified by PA assessment methods. In addition, the heterogeneity among the existing studies in the selection of walking performance assessment indicators (e.g., frequency, intensity, etc.) and the measurement of the contextual factors has limited the comparison between studies.

To date, no standardized classification method for contextual factors has been established. A comprehensive classification framework was proposed by Chen (15), which categorizes contextual factors into user context, environmental context, and task context based on the requirements of context-aware technology and a user-centered approach. The user context dimension includes user attributes, living habits, and behavioral habits. The environmental context dimension includes the natural physical environment, comprising time, location, sunlight, and social environment (national customs and religious habits). Task context refers to the attributes and states of the behaviors associated with the user’s completion of the current task. The framework may provide a theoretical basis for the integration of multi-source contextual factors. Therefore, this study aimed to synthesize and categorize the contextual factors influencing walking performance in individuals with stroke, drawing on the contextual factor classification framework proposed by Chen (15) and to identify through quantitative synthesis those contextual factors demonstrating significant associations with walking performance. These efforts seek to provide a theoretical foundation for context-aware intervention development. Additionally, the study aimed to describe walking performance in individuals with stroke using real-world data, thereby updating the current status of walking performance among patients with stroke.

## 2 Methods

### 2.1 Registration

This review was registered on PROSPERO (CRD420250651772).

### 2.2 Eligibility criteria

The inclusion criteria were as follows (1): the study population diagnosed with a stroke (either ischemic, hemorrhagic, or a combination of both) (2); studies assessing walking performance in real-world settings (i.e., non-clinical environments excluding hospitals and laboratories) must include at least one of the following objective metrics: daily step count, walking duration, stepping frequency, or walking distance (3); studies that concurrently investigate walking performance and contextual factors and that provide empirical evidence of the correlation between contextual factors and walking performance metrics or the causal effect of contextual factors on walking performance (4); observational study design. The exclusion criteria were as follows (1): duplicate publication of study data (2); conference papers, reviews, protocols, randomized controlled trials or meta-analyses (3); articles without available full text; and (4) non-English articles.

### 2.3 Information sources

From the inception to September 4, 2024, a systematic search was conducted across 4 databases, including the Cochrane Library, PubMed, Web of Science, and Embase.

### 2.4 Search strategy

The search keywords were expanded to movement behavior, including physical activity and sedentary behavior, to ensure the comprehensiveness of the literature search. The search terms were tailored to individual databases. Please refer to Supplementary Table 1 for detailed search strategies. Only publications written in English were included. In addition, the reference lists of included articles were screened to identify potential studies.

### 2.5 Selection process

We imported the identified records into EndNote X9 and deleted duplicate records. Two researchers independently screened the identified articles based on the inclusion and exclusion criteria. Differences were resolved via discussion or consultation with other researchers.

### 2.6 Data extraction

We extracted and recorded the data using a homemade Excel spreadsheet. We extracted author names, publication date, country of study, study design, sample size, participant characteristics (including age, sex, stroke severity, and time since stroke), walking performance (daily step count, walking duration, walking distance, etc.), measurement tools, the contextual factors investigated, statistical tests employed for analysis, and correlation values. Two researchers independently extracted information and cross-checked the data. Differences in opinion were judged via discussion or via the participation of other researchers. For missing data, we contacted study investigators to obtain unreported data or additional details.

### 2.7 Risk of bias assessment

Cross-sectional and longitudinal studies were assessed for bias using the 11 evaluation criteria recommended by the Agency for Healthcare Research and Quality (AHRQ) (16). Each of the 11 assessment criteria recommended by the AHRQ received “yes” (1 point), “no” (0 points), or “unclear” (0 points). The total score was 11 points, with 0–3 indicating low quality, 4–7 indicating moderate quality, and 8–11 indicating high quality. The assessment was independently conducted by two researchers, and a third researcher was consulted in cases of uncertainty or disagreement.

### 2.8 Strategy for data synthesis

Guided by the context-aware classification framework, two researchers independently performed classification on the contextual factors extracted from the original studies. Upon completion of the classification process, a cross-check of their respective work was conducted to identify any discrepancies. All inconsistencies were subjected to thorough deliberation with the aim of reaching mutual agreement; in instances where consensus remained elusive, a third researcher was consulted to render the final decision.

The meta-analysis was conducted using Stata 17.0 software. Quantitative synthesis was conducted when two or more studies reported walking performance outcomes or correlations between walking performance and situational factors. Weighted means (by sample size) and 95% CIs were calculated using Stata 17.0 for each walking performance outcome. Based on the methods proposed by Luo et al. (17) and Wan et al. (18), we transformed medians, ranges, or quartiles into mean ± standard deviation. Regarding contextual factors, the correlation values (*r*) were used to calculate the effect size (ES). The formula was employed to convert r to Fisher’s *z*-value with standard error, and finally converted the Fisher’s *z*-value back to *r*. For standardized regression coefficients or path coefficient analyses, the formula *r* = 0.98*β* + 0.05*λ* (*λ* = 1 when *β* ≥ 0; *λ* = 0 when *β* < 0) was utilized to transform the regression coefficients into correlation coefficients (*r*) before subsequent calculations (19). First, the correlational (*r*) values were converted to (ES(*z*) = 1/2log_e_ [(1 + *r*)/(1 − *r*)]) (20), and then the pooled effect size was calculated. Statistical heterogeneity was judged based on *p*-value and *I*^2^. *I*^2^ ≤ 50% or *p* ≥ 0.05 indicated acceptable heterogeneity among studies; thus, a fixed-effects model was employed for analysis, otherwise a random-effects model was used for analysis. Subgroup analysis and meta-regression were performed to analyze the source of heterogeneity when heterogeneity was significant. Pooled ES was converted back to r using meta *r* = ([e^(2z)^ − 1]/[e^(2z)^ + 1]) (20). Correlation coefficient (*r*) < 0.1 indicated no relationship, *r* between 0.1 and 0.29 indicated a weak relationship, r between 0.30 and 0.49 indicated a moderate relationship, and *r* ≥ 0.5 indicated a strong relationship (21). Sensitivity analysis was performed by excluding the included literature one by one to evaluate the stability of the meta-analysis results. When at least 10 studies were included, publication bias was assessed using a funnel plot.

## 3 Results

### 3.1 Study selection and characteristics

**Figure 1:**
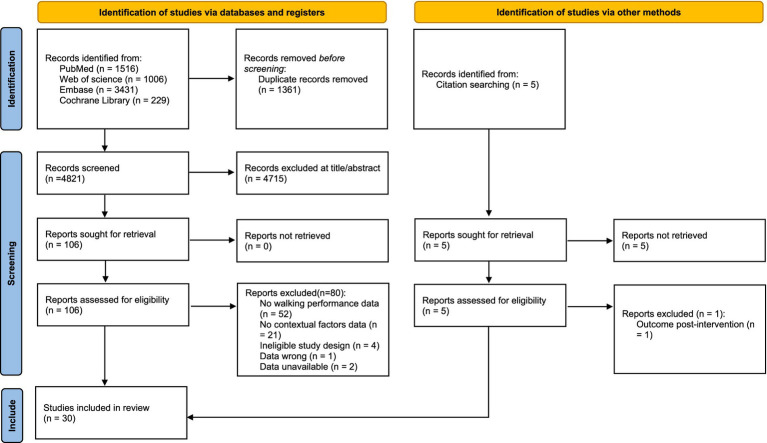
Flow chart of study selection.

The database search initially yielded 6,182 potential articles, from which 1,361 duplicates were excluded. After screening the titles and abstracts of the remaining 4,821 articles, we excluded 4,715 irrelevant articles. Full-text reports of 106 studies were assessed for eligibility, of which 26 met the criteria and were included in the synthesis. We reviewed the reference lists of the 26 included studies and identified five additional studies that met the eligibility criteria. In total, 30 studies published between 2005 and 2024 were included in our study. The detailed search process is depicted in Figure 1.

The extracted data are summarized in Supplementary Table 2. Most studies had a cross-sectional design, and 4 studies were longitudinal. In total, 2,217 patients with stroke were enrolled in 30 studies, of which 1,283 (57.9%) were male. One study did not report sex. Nine studies reported stroke severity using the National Institutes of Health Stroke Scale, with most patients experiencing stroke with low or moderate severity. Participants ranged from 3 months to 14 years post-stroke. Seven studies did not report time since stroke.

### 3.2 Risk of bias in studies

Based on the AHRQ items, the quality assessment revealed that 20 studies showed moderate quality and 10 studies exhibited high quality (Supplementary Table 3).

### 3.3 Measurement tools and indicators of walking performance

Walking performance was measured using Activity Monitor, most commonly the StepWatch Activity Monitor (SAM) (22–28) and Fitbit family (Fitbit One, Fitbit Zip, and Fitbit Charge HR) (29–36). Other devices included activPAL^™^ (37, 38), activPAL (39, 40), activPAL3 Micro (41), Actigraph (42), Actigraph GT3X (43), tri-axial accelerometer (44), hip accelerometer (45), Yamax SW-200 pedometer (46), VKRFitness Twin Step Pedometer (47), OMRON step Counter (48), SenseWear Armband (49, 50), and SenseWear Pro 3 Armband (51).

Step count was the most commonly applied indicator of walking performance. Other indicators were time in stepping (38, 39, 41, 44), walking distance (48), and walking bouts (40, 44). Seven studies stratified walking performance to intensities (23, 24, 27, 37, 38, 40, 41), and three studies stratified walking performance to time of walking bouts (37, 40, 44). However, there was no consensus on the definition of walking intensity. Two studies defined low-intensity walking as less than 16 steps per minute (23, 27), while one study defined low-intensity walking as less than 30 steps per minute (24). Additionally, two other studies defined low-intensity walking as less than 80 steps per minute (38, 41). Different studies offered different definitions of medium-intensity walking. One study defined medium-intensity walking as ≥16 and <30 steps per minute (27), another defined medium-intensity walking as 16–40 steps per minute (23), and another study defined medium-intensity walking as 80–99 steps per minute (38, 41). Different studies defined high-intensity as ≥30 steps per minute (27), >40 steps/min (23), >60 steps/min (24), >80 steps/min (37), and >100 steps/min (38, 41). In addition, Luzum et al. (40) utilized metabolic equivalent of task (MET) to classify walking intensity.

There were also differences in the definition of walking bouts in different studies. van de Port et al. (44) defined a walking bout as an episode of one or more consecutive gait epochs of 8 s and classified them into short walking bouts (8–24 s) and long walking bouts (>24 s). Mahendran et al. (37) defined a walking bout as any 15-s data interval containing 2 steps and defined long walking bouts as >300 steps. Luzum et al. (40) defined standing and walking events as a minimum length of 3 s. Events lasting from 3 s to less than 10 min were categorized as short-bout PA, while those lasting 10 min or more were classified as long-bout PA.

### 3.4 Contextual factors model of walking performance in patients with stroke

**Figure 2:**
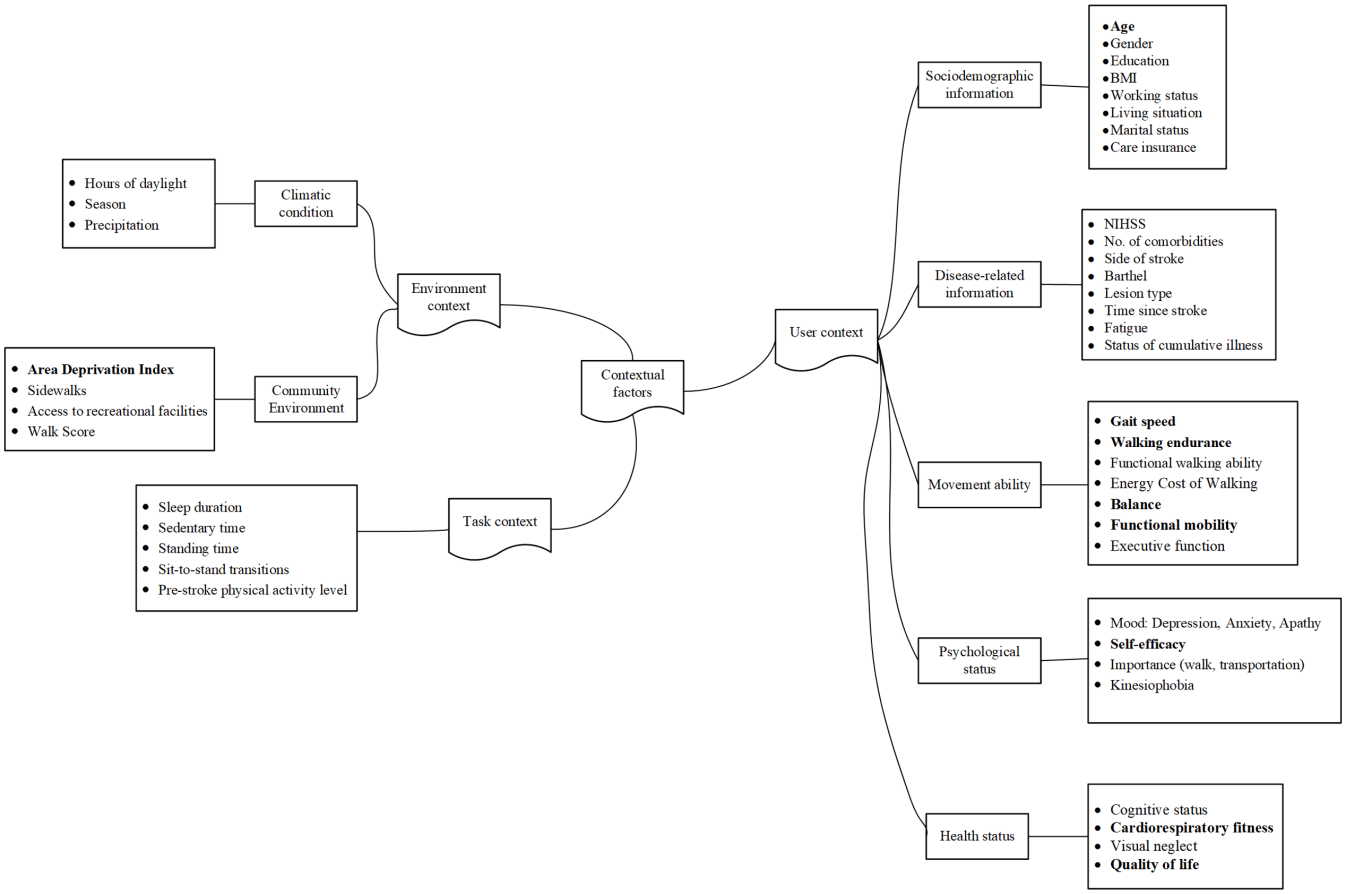
A contextual factor classification model for the walking performance of patients with stroke.

We extracted contextual factors associated with walking performance from the 30 included studies and categorized them based on the contextual factors classification model (15). Our model (Figure 2) was composed of three essential dimensions: user context, environment context, and task context. The user context involved demographic information, disease-related information, movement capacity, psychological status, and health status of patients with stroke. Environmental contexts included climatic conditions and community environments. Task contexts are other movement behavioral states closely associated with walking activity, including physical activity, sedentary behavior, standing, and sit-to-stand transitions.

### 3.5 Synthesis of meta-analysis results

#### 3.5.1 Pooled daily steps

**Figure 3:**
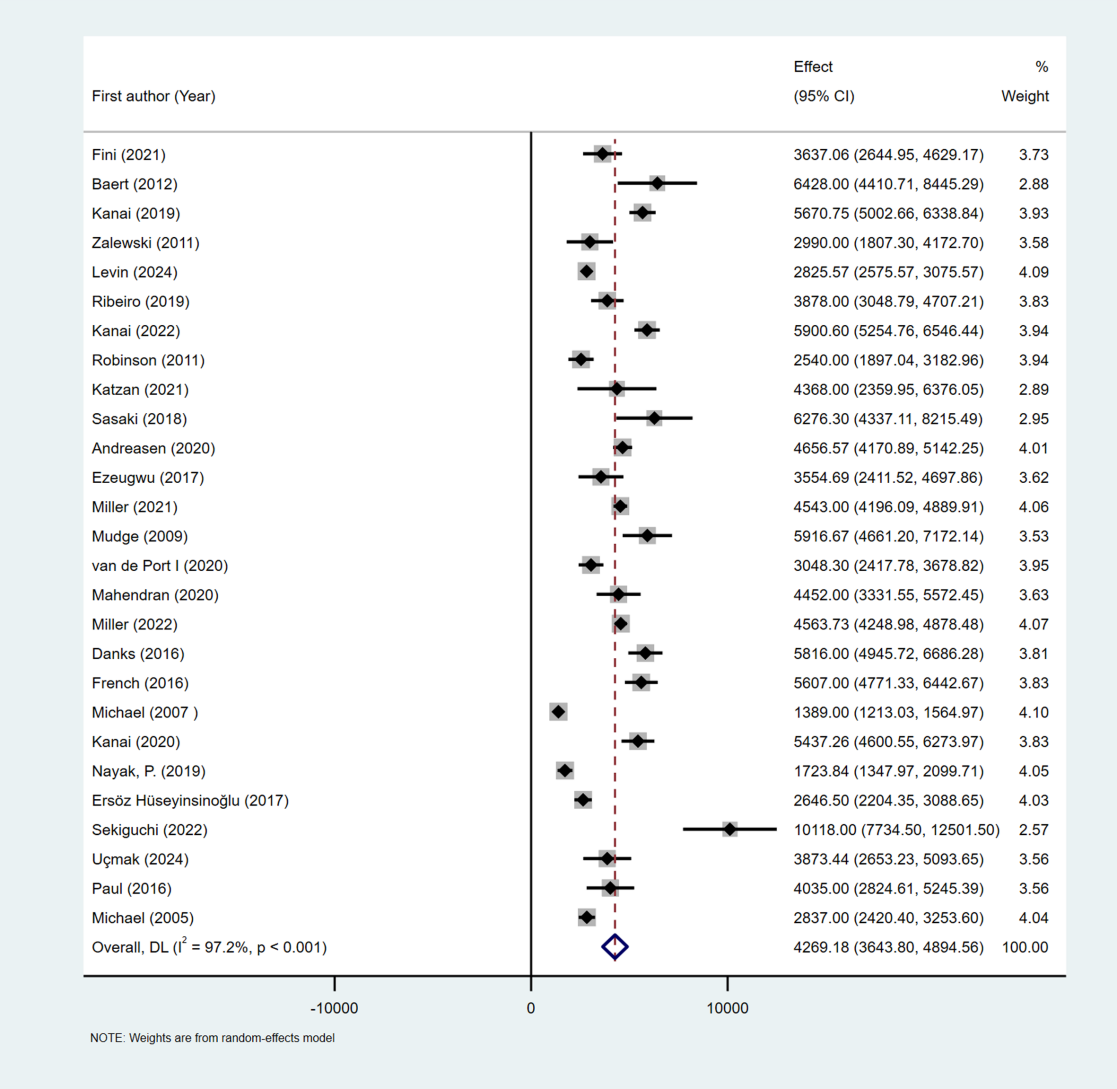
Forest plots of the pooled outcome with corresponding 95% CIs for daily steps of patients with stroke.

Due to inconsistent reporting of walking performance metrics across studies, daily step count was selected as the indicator for quantitative synthesis, as it was the most consistently reported and standardized metric. Twenty-seven of the included articles reported daily steps as an outcome indicator. Meta-analysis revealed that the number of daily steps of patients with stroke in a free-living environment was 4,269 (95% CI: 3643.80 to 4894.56), with significant heterogeneity among the included studies (*I*^2^ = 97.2%, *p* < 0.001); therefore, the random-effects model was employed (Figure 3).

#### 3.5.2 Subgroup analysis and meta-regression of pooled daily step counts

The subgroups were grouped based on the year of publication, sample size, time since stroke, region, and measurement instrument. There was significant heterogeneity between the subgroups (*I*^2^ > 50% or *p* < 0.05); thus, they were analyzed using a random-effects model. Except for the subgroups of sample size and measurement tools, there was no significant difference between subgroups (*p* > 0.05) (Supplementary Table 4).

Subgroup analysis based on the year of publication indicated that the number of steps per day (95% CI) was 3,977 (3135.431–4819.523) for the 2005–2019 subgroup and 4,563 (3871.890–5253.761) for the 2020–2024 subgroup, respectively. The subgroups were categorized based on sample size into three groups: <30, 30–49, and ≥50, and significant differences were observed in the number of steps between the subgroups (*p* = 0.01). The respective combined daily step counts (95% CI) for these subgroups were 5,211 (3827.281–6596.278), 3,215 (2642.331–3786.923), and 4,538 (3360.859–5714.966), respectively. In subgroup analysis based on region, the daily step count was 4,248 (3062.355–5433.143) in Europe, 4,680 (3565.475–5794.810) in Asia, 3,881 (2848.232–4914.365) in America, and 4,743 (2510.074–6976.063) in Australia. The number of steps taken per day by elderly patients with stroke (aged 65 years and older) was 4,231 (2999.001–5464.547), while the number of steps was 4,251 (3590.427–4912.523) for patients with stroke who were younger than 65 years. The population was divided into three subgroups based on time since stroke, including less than 3 months, 3–6 months, and more than 6 months. Daily steps for these subgroups were 3,217 (1628.817–4805.504), 4,729 (3971.831–5486.925), and 4,196 (3445.838–4946.693), respectively. When studies were divided into 4 subgroups based on the measurement tool, there was a significant difference in the number of daily steps between the groups (*p* = 0.003). Studies using the Fitbit family as the measurement tool had a combined daily step count of 5,076 (4647.491–5504.303), significantly higher than that in studies using other measurement tools. Meta-regression did not identify significant sources of heterogeneity (see Supplementary Table 5).

#### 3.5.3 Contextual factors associated with daily steps

After a thorough examination of the full-text version of the 30 articles, 4 were excluded due to insufficient information necessary for calculating the correlation coefficients (25, 34, 45, 50). Additionally, three articles investigated the associations between walking time and contextual factors, yet these factors did not overlap with others, leading to their exclusion (38–40). Furthermore, the contextual factors explored in the two articles were not replicated in the broader literature, necessitating their exclusion (23, 42). The remaining studies containing the same contextual factors were pooled (≥2 studies), with the standardized regression coefficients reported in seven of these studies converted to correlation coefficients using the formula (28, 29, 31, 32, 35, 44, 51). Table 1 reveals the pooled r value obtained via *z*-to-*r* back transformation.

**Table 1:** Results of meta-analysis for contextual factors of walking performance among individuals with stroke.

Contextual factors	Number of studies included	Meta-analysis		
		*r*	95% CI low	95% CI upper
Age	9 (24, 29, 32, 35, 37, 41, 46–48)	−0.10	−0.18	−0.02
Gender	4 (29, 32, 35, 46)	−0.08	−0.18	0.02
Time since stroke	2 (41, 48)	−0.21	−0.45	0.05
Fatigue	5 (27, 28, 37, 47, 51)	−0.11	−0.34	0.14
Cognitive status	2 (41, 48)	0.07	−0.20	0.33
Quality of life	7 (33, 36, 37, 41, 43, 46, 51)	0.46	0.34	0.56
Gait speed	10 (22, 24, 26, 29, 30, 35, 37, 41, 44, 46)	0.41	0.23	0.56
Walking endurance	4 (22, 24, 30, 37)	0.60	0.46	0.71
Area deprivation index	2 (31, 32)	−0.15	−0.24	−0.06
Depression	6 (28, 37, 44, 46–48)	−0.09	−0.32	0.16
Cardiorespiratory fitness	4 (26, 27, 46, 49)	0.36	0.09	0.59
Balance ability	3 (26, 30, 44)	0.37	0.02	0.65
NIHSS	2 (29, 35)	−0.01	−0.18	0.16
Economy of gait	2 (26, 27)	−0.06	−0.24	0.11
Self-efficacy	4 (28, 31, 37, 47)	0.30	0.21	0.39
RMA	2 (24, 46)	0.49	0.26	0.65

NIHSS, National Institutes Hospital Stroke Scale; RMA, Rivermead Motor Assessment; CI, confidence interval.

##### 3.5.3.1 User context

###### 3.5.3.1.1 Demographic information

**Figure 4:**
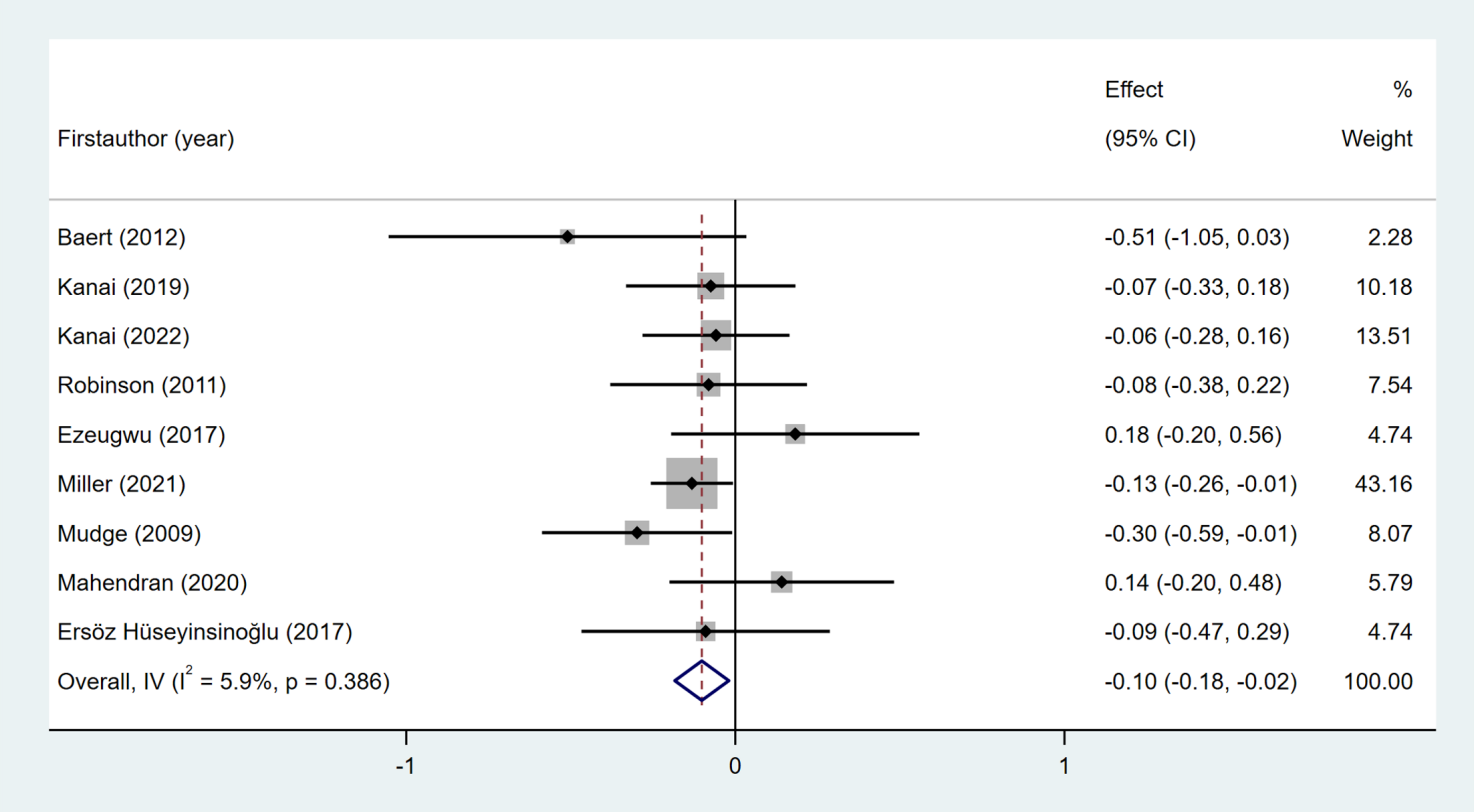
Forest plot for the relationship between age and daily steps.

**Figure 5:**
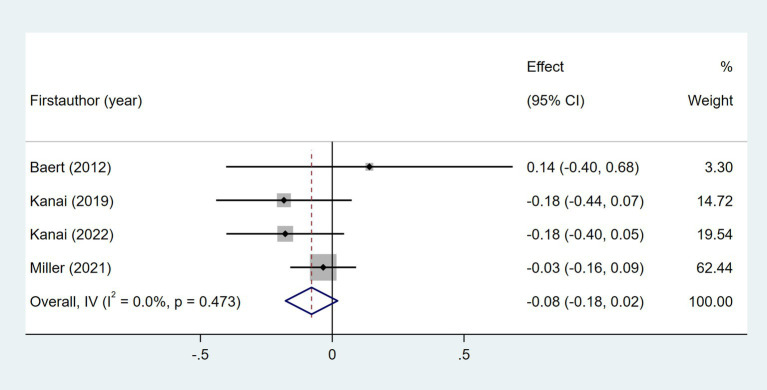
Forest plot for the relationship between gender and daily steps.

Nine studies examined the relationship between age and daily steps (24, 29, 32, 35, 37, 41, 46–48). As shown in Figure 4, meta-analysis revealed a significant but weak relationship between post-stroke daily steps and age (Fisher’s *z* = −0.1; 95% CI, −0.18 to −0.02; meta *r* = −0.10; 95% CI, −0.18 to −0.02). Four studies investigated the relationship between gender and daily steps (29, 32, 35, 46). As shown in Figure 5, the meta-meta-analysis revealed no statistically significant association (Fisher’s *z* = −0.08; 95% CI, −0.18 to 0.02; meta *r* = −0.08; 95% CI, −0.18 to 0.02).

###### 3.5.3.1.2 Disease-related information

**Figure 6:**
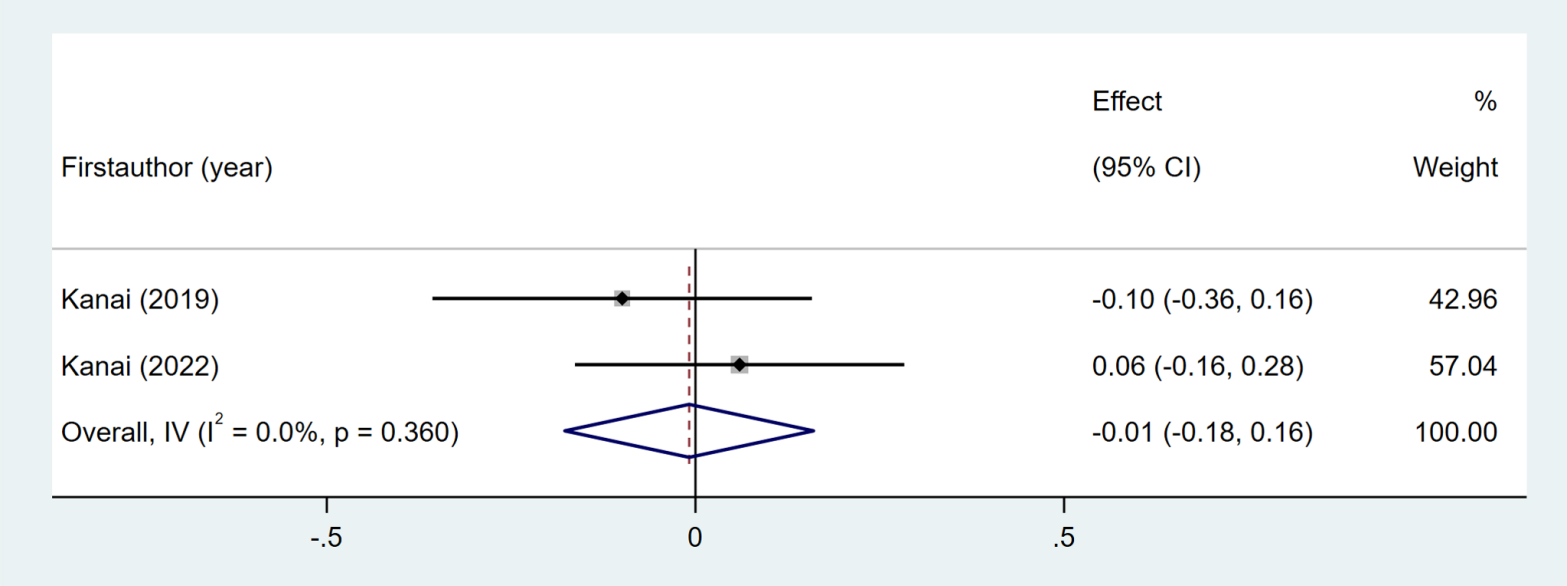
Forest plot for the relationship between NIHSS and daily steps.

**Figure 7:**
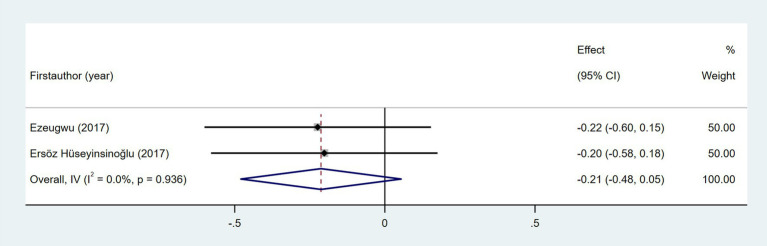
Forest plot for the relationship between time since stroke and daily steps.

**Figure 8:**
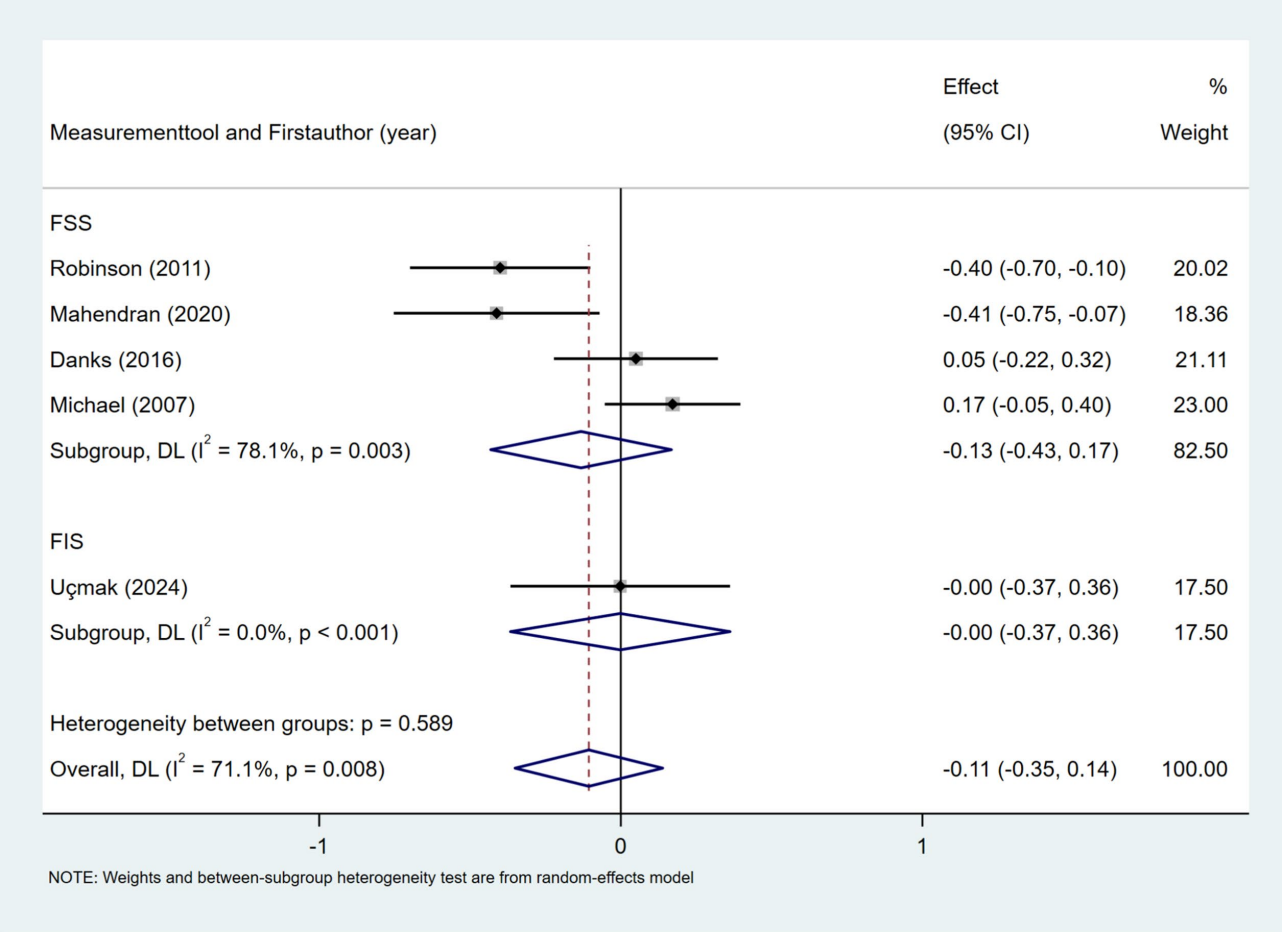
Forest plot for the relationship between fatigue and daily steps. FSS, Fatigue Severity Scale; FIS, Fatigue Impact Scale.

The relationship between NIHSS score and daily steps was investigated in two studies (29, 35). As shown in Figure 6, the meta-analysis revealed no statistically significant association (Fisher’s *z* = −0.01; 95% CI, −0.18 to 0.16; meta *r* = −0.01; 95% CI, −0.18 to 0.16). As shown in Figure 7, the meta-analysis of two studies revealed no significant association between time since stroke and daily step count (Fisher’s *z* = −0.21; 95% CI, −0.48 to 0.05; meta *r* = −0.21; 95% CI, −0.45 to 0.05). Five studies explored the association between fatigue and daily step count (27, 28, 37, 47, 51). Fatigue was assessed using validated instruments including the Fatigue Severity Scale (FSS) and the Fatigue Impact Scale (FIS). As shown in Figure 8, the meta-analysis revealed no statistically significant association between fatigue and daily step count (Fisher’s *z* = −0.11; 95% CI, −0.35 to 0.14; meta *r* = −0.11; 95% CI, −0.34 to 0.14), but substantial heterogeneity was observed (*I*^2^ = 71.1%, *p* = 0.008). Subgroup analysis demonstrated that measurement tools did not significantly account for the observed heterogeneity across studies (*p* = 0.589).

###### 3.5.3.1.3 Movement capacity

**Figure 9:**
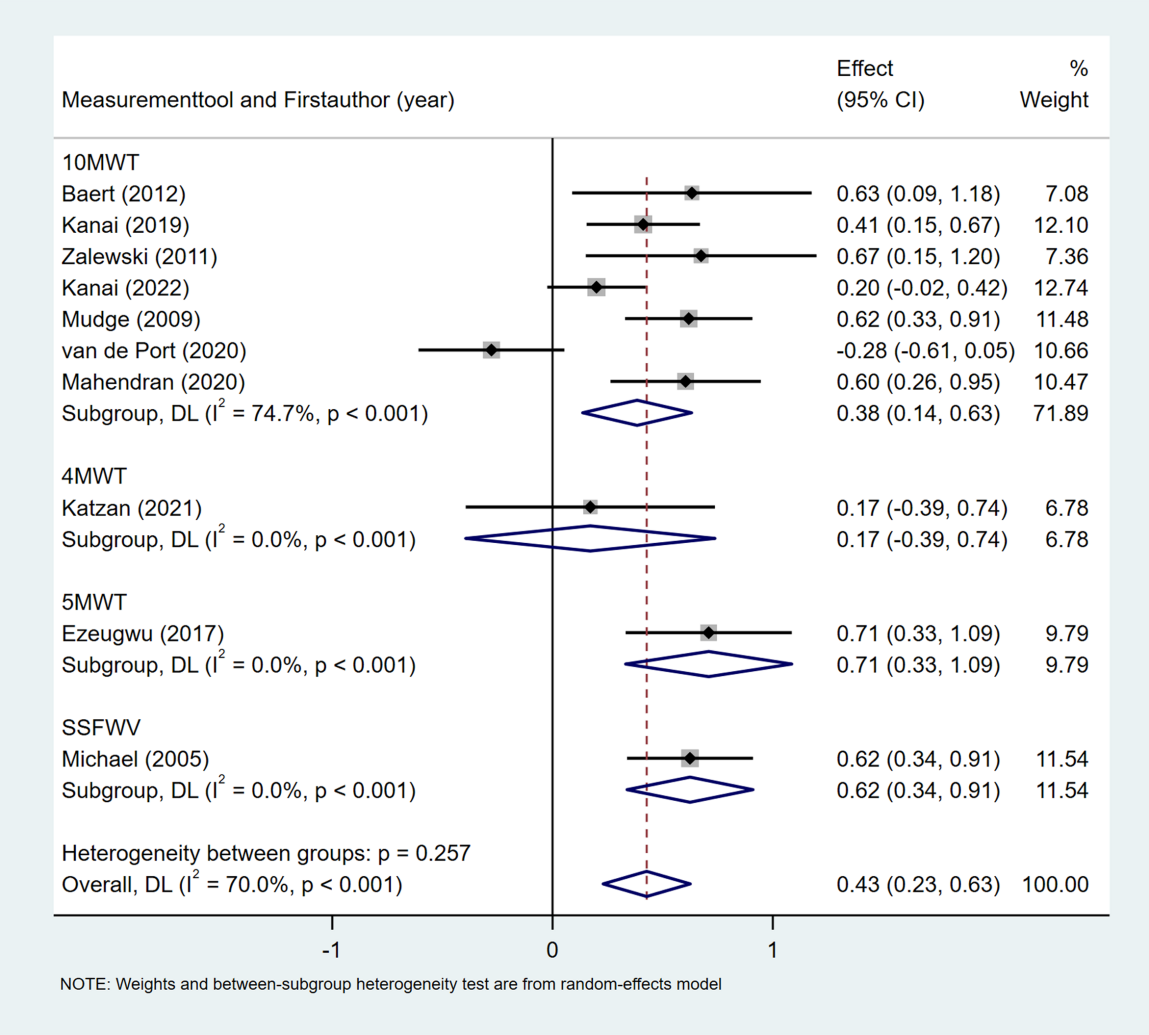
Forest plot for the relationship between gait speed and daily steps. 10MWT, 10 Meter Walk Test; 4MWT, 4 Meter Walk Test; 5MWT, 5 Meter Walk Test; SSFWV, self-selected floor walking velocity.

**Figure 10:**
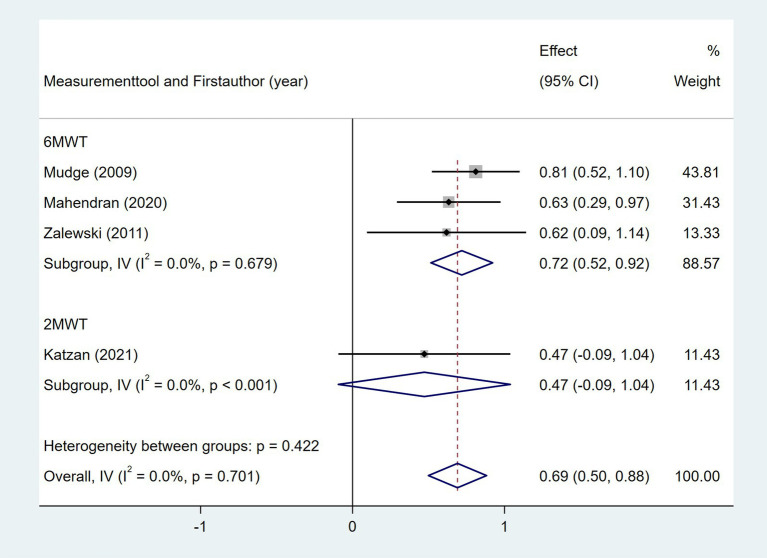
Forest plot for the relationship between walking endurance and daily steps. 6MWT, 6-Minute Walk Test; 2MWT, 2-Minute Walk Test.

**Figure 11:**
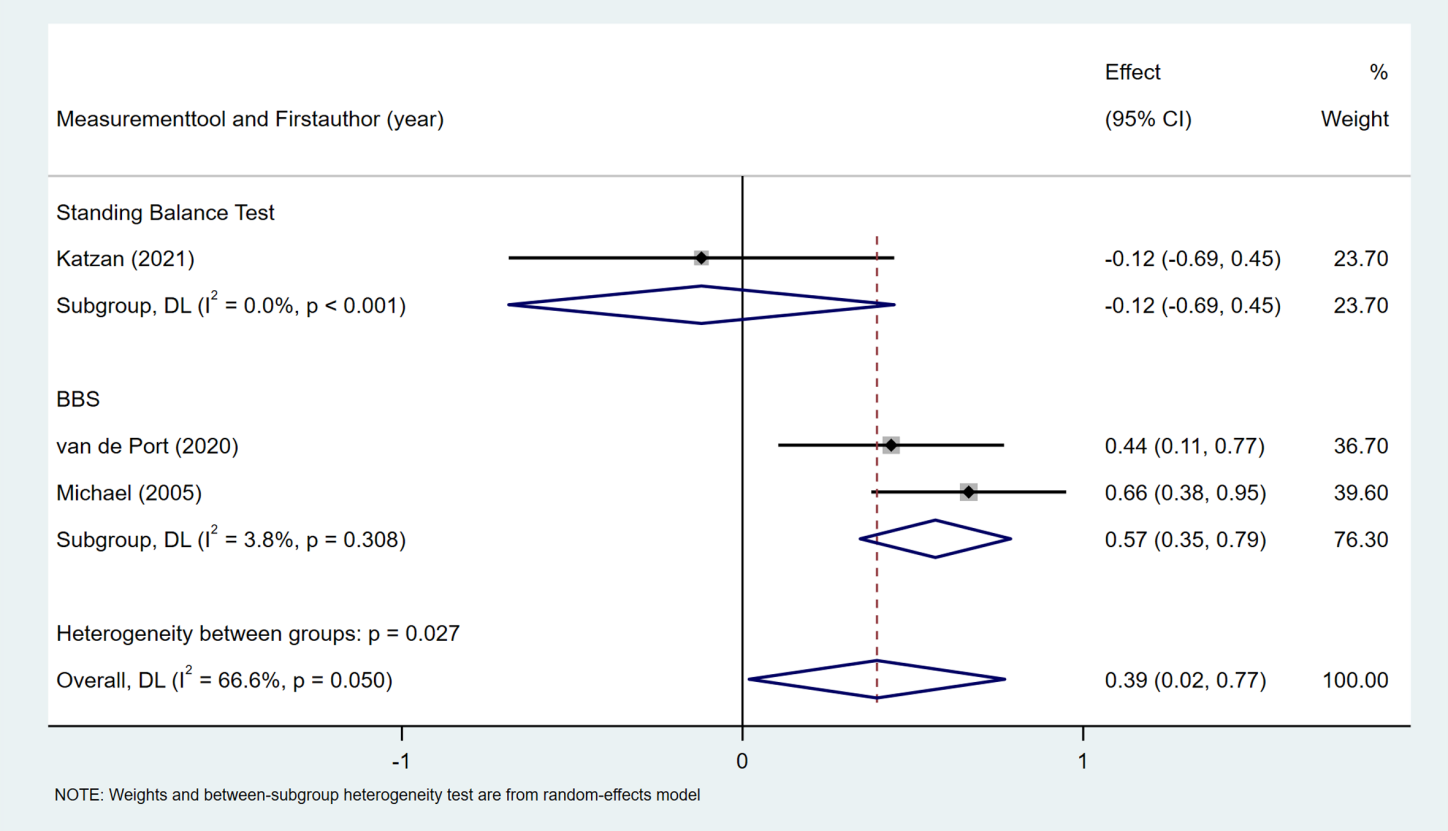
Forest plot for the relationship between balance and daily steps. BBS, Berg Balance Scale.

**Figure 12:**
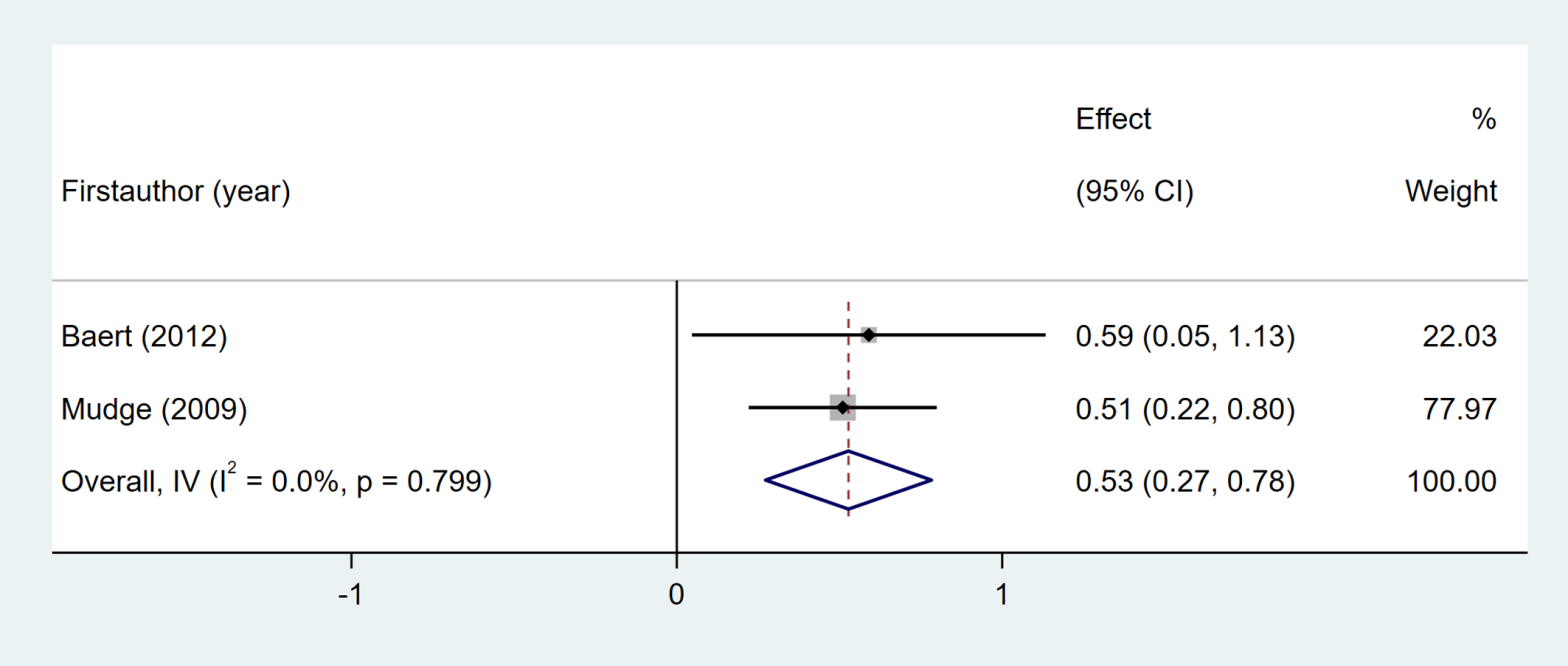
Forest plot for the relationship between RMA and daily steps. RMA, Rivermead Motor Assessment.

**Figure 13:**
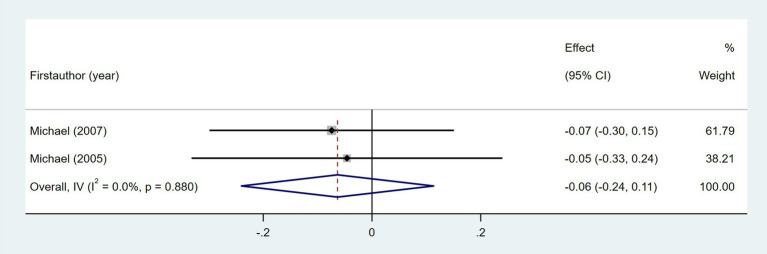
Forest plot for the relationship between economy of gait and daily steps.

Ten studies investigated the relationship between gait speed and daily step count, employing diverse methods to assess gait speed: 10 Meter Walk Test (10MWT) (22, 24, 29, 35, 37, 44, 46), 4 Meter Walk Test (4MWT) (30), 5 Meter Walk Test (5MWT) (41), and self-selected floor walking velocity (26). In Figure 9, the meta-analysis revealed a moderate positive correlation between gait speed and daily step count (Fisher’s *z* = 0.43; 95% CI, 0.23 to 0.63; meta *r* = 0.41; 95% CI, 0.23 to 0.56). Moreover, between-study heterogeneity was high (*I*^2^ = 70.0%, *p* < 0.001); between-study heterogeneity was largely attributed to a single study that demonstrated a negative association (44), while measurement tools did not significantly explain the observed heterogeneity (*p* = 0.257). Four studies investigated the association between walking endurance and daily step count, using the 6-Minute Walk Test (6MWT) (22, 24, 37) and the 2-Minute Walk Test (2MWT) (30). As shown in Figure 10, the meta-analysis demonstrated a strong, statistically significant positive correlation between walking endurance and daily step count in patients with stroke (Fisher’s *z* = 0.69; 95% CI, 0.50 to 0.88; meta *r* = 0.60; 95% CI, 0.46 to 0.71). Three studies investigated the association between balance and daily step count in patients with stroke using the Berg Balance Scale (BBS) (26, 44) and the standing balance test (30). As shown in Figure 11, the meta-analysis revealed a moderate correlation (Fisher’s *z* = 0.39; 95% CI, 0.02 to 0.77; meta *r* = 0.37; 95% CI, 0.02 to 0.65), with high heterogeneity (*I*^2^ = 66.6%, *p* = 0.05). Balance assessment tools can identify subgroup variations, thereby addressing potential sources of heterogeneity in pooled effect estimates (*p* = 0.027). The Rivermead Motor Assessment (RMA) is an important measure of mobility in patients with stroke. Two studies evaluated the relationship between the RMA and daily step count (24, 46), with the meta-analysis of these studies confirming a moderate correlation (Fisher’s *z* = 0.53; 95% CI, 0.27 to 0.78; meta *r* = 0.49; 95% CI, 0.26 to 0.65) (Figure 12). Two studies investigated the association between economy of gait and daily step count (26, 27). As shown in Figure 13, the meta-analysis revealed no statistically significant correlation (Fisher’s *z* = −0.06; 95% CI, −0.24 to 0.11; meta *r* = −0.06; 95% CI, −0.24 to 0.11).

###### 3.5.3.1.4 Psychological status

**Figure 14:**
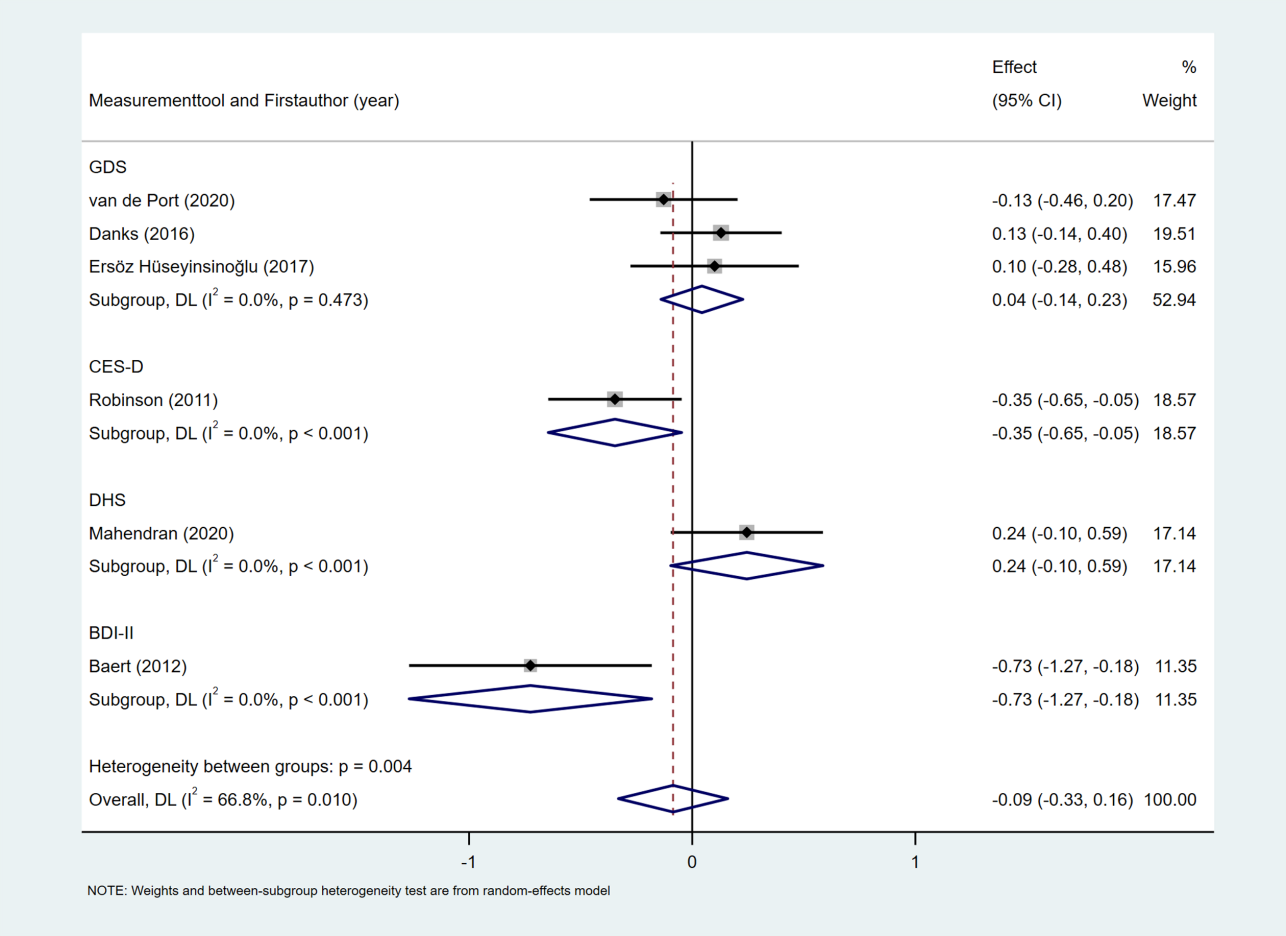
Forest plot for the relationship between depression and daily steps. GDS, Geriatric Depression Scale; CES-D, Center for Epidemiologic Studies Depression Scale; DHS, Depression-Happiness Scale; BDI-II, Beck Depression Inventory-II.

**Figure 15:**
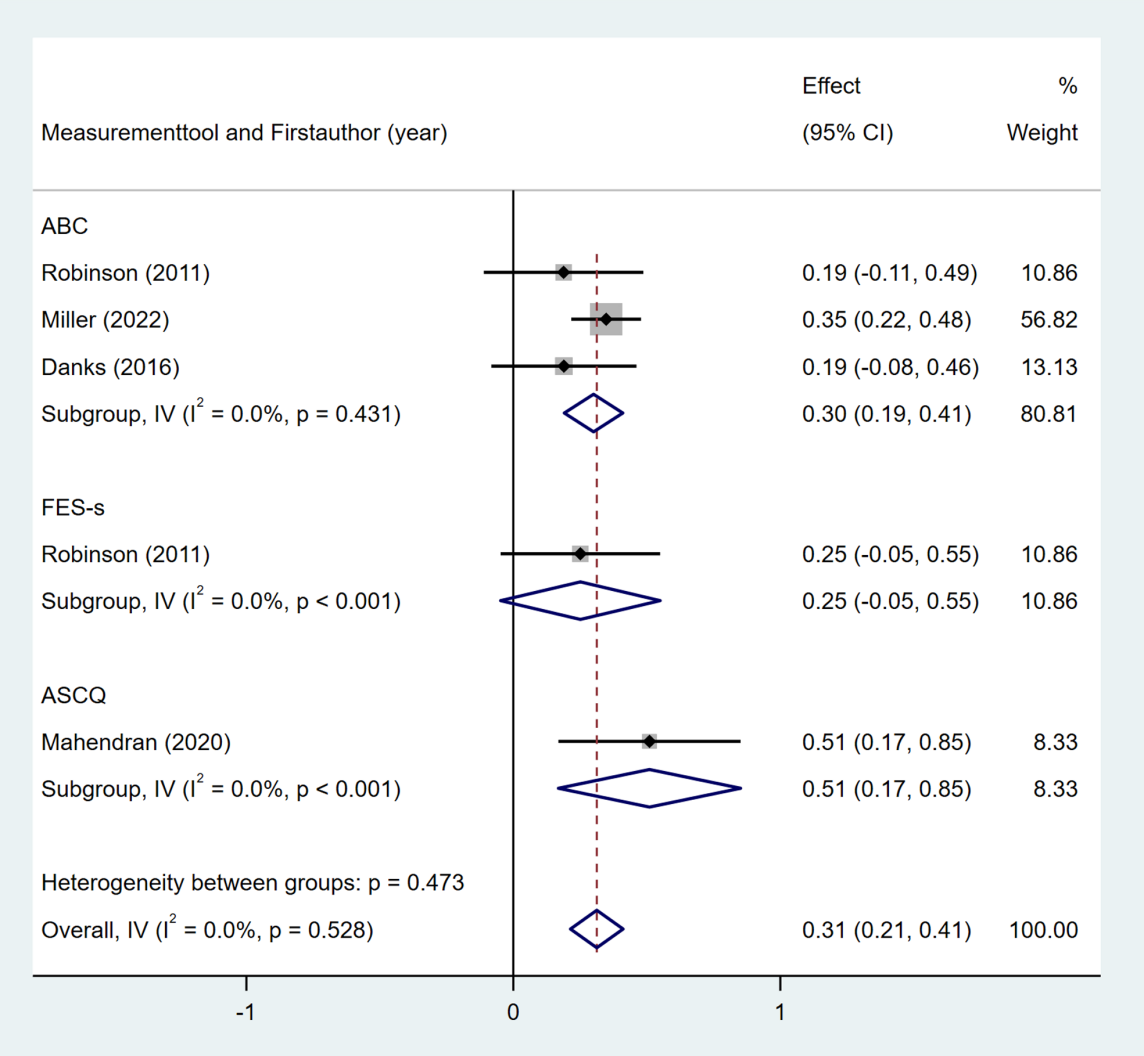
Forest plot for the relationship between self-efficacy and daily steps. ABC, Activities Specific Balance Confidence Scale; FES-s, Swedish version of the Falls Efficacy Scale; ASCQ, Ambulatory Self-Confidence Questionnaire.

Six studies explored the association between depression and daily step count (28, 37, 44, 46–48), utilizing validated depression scales including the Geriatric Depression Scale (GDS), Beck Depression Inventory-II (BDI-II), Center for Epidemiologic Studies Depression Scale (CES-D), and Depression-Happiness Scale (DHS). As shown in Figure 14, random-effects meta-analysis of six studies demonstrated a non-significant correlation between depression and objectively measured daily steps (Fisher’s *z* = −0.09; 95% CI, −0.33 to 0.16; meta *r* = −0.09; 95% CI, −0.32 to 0.16). However, substantial heterogeneity was observed (*I*^2^ = 66.8%, *p* = 0.01). Subgroup analyses indicated that heterogeneity was attributable to variations in depression assessment tools (*p* = 0.004). Four studies investigated the association between self-efficacy and daily step count, with self-efficacy assessed using validated instruments including the Activities Specific Balance Confidence Scale (ABC) (28, 31, 47), the Ambulatory Self-Confidence Questionnaire (ASCQ) (37), and the Swedish version of the Falls Efficacy Scale (FES-s) (47). As shown in Figure 15, the meta-analysis revealed a moderate positive correlation between self-efficacy and daily step count (Fisher’s *z* = 0.31; 95% CI, 0.21 to 0.41; meta *r* = 0.30; 95% CI, 0.21 to 0.39).

###### 3.5.3.1.5 Health status

**Figure 16:**
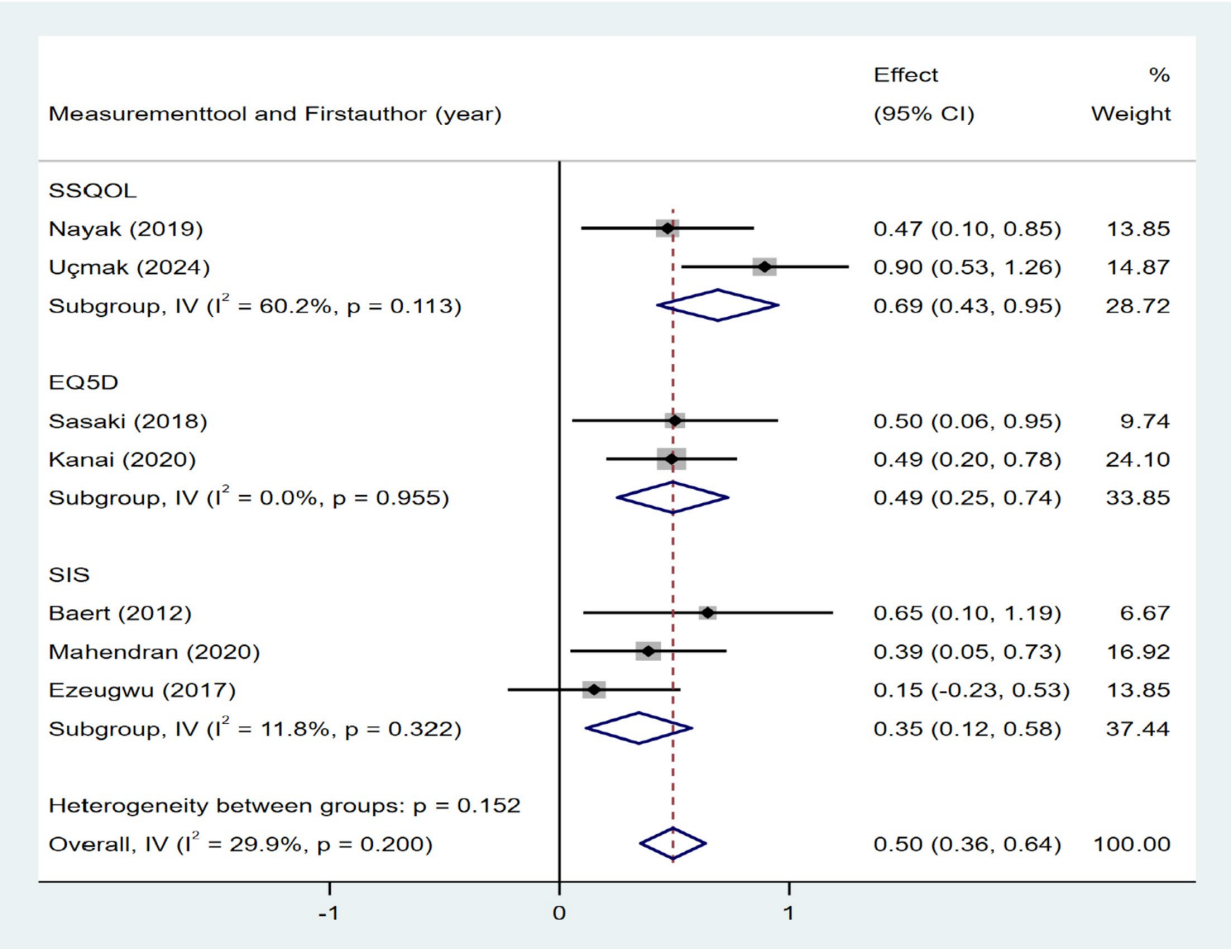
Forest plot for the relationship between quality of life and daily steps. SSQOL, Stroke-Specific Quality of Life Scale; EQ5D, EuroQol Five-Dimensions; SIS, Stroke Impact Scale.

**Figure 17:**
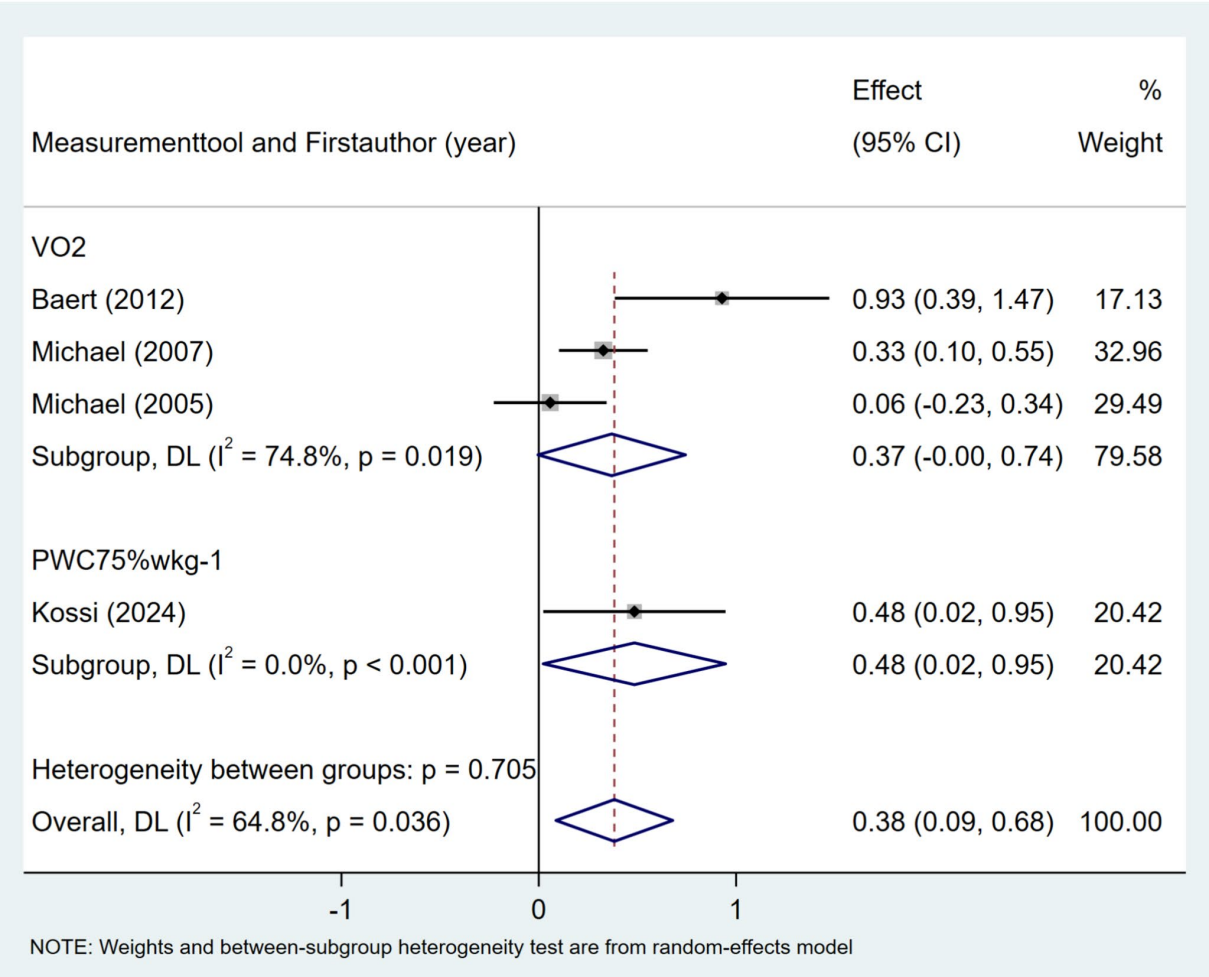
Forest plot for the relationship between cardiorespiratory fitness and daily steps.

**Figure 18:**
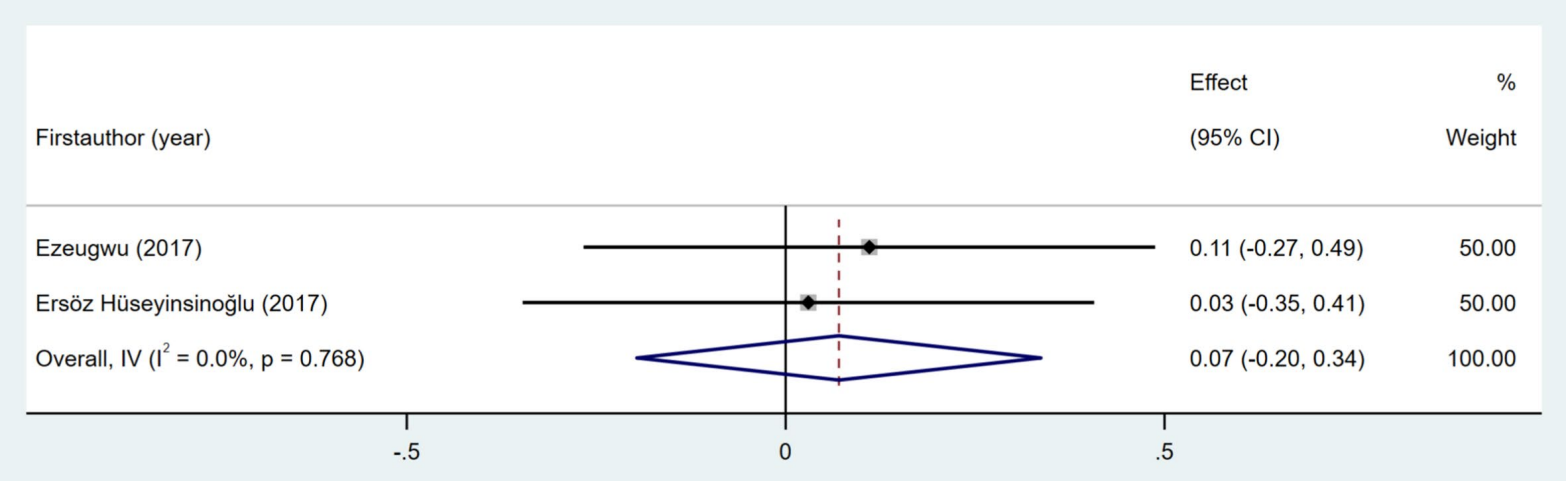
Forest plot for the relationship between cognitive status and daily steps.

Seven studies investigated the association between quality of life and daily step count. The quality of life was assessed using the Stroke-Specific Quality of Life Scale (SSQOL) (43, 51), Stroke Impact Scale (SIS) (37, 41, 46), and EuroQol Five-Dimensions (EQ5D) (33, 36). As shown in Figure 16, the meta-analysis revealed a moderate positive correlation between quality of life and daily step count (Fisher’s *z* = 0.5; 95% CI, 0.36 to 0.41; meta *r* = 0.64; 95% CI, 0.34 to 0.56). Cardiorespiratory fitness, measured based on peak oxygen consumption (VO_2_ peak) (26, 27, 46) or the physical working capacity at 75% of the predicted maximal heart rate/kilogram of body weight (PWC_75%_ w kg^−1^) (49), was investigated in four studies. As shown in Figure 17, the meta-analysis revealed a moderate positive correlation between cardiorespiratory fitness and daily step count (Fisher’s *z* = 0.38; 95% CI, 0.09 to 0.68; meta *r* = 0.36; 95% CI, 0.09 to 0.59). However, substantial heterogeneity was observed (*I*^2^ = 64.8%, *p* = 0.036), although measurement tools did not significantly contribute to this variability (*p* = 0.705). Two studies investigated the relationship between cognitive status and daily step count (41, 48). As shown in Figure 18, the meta-analysis found no statistically significant association (Fisher’s *z* = 0.07; 95% CI, −0.2 to 0.34; meta *r* = 0.07; 95% CI, −0.2 to 0.33).

##### 3.5.3.2 Environmental context

**Figure 19:**
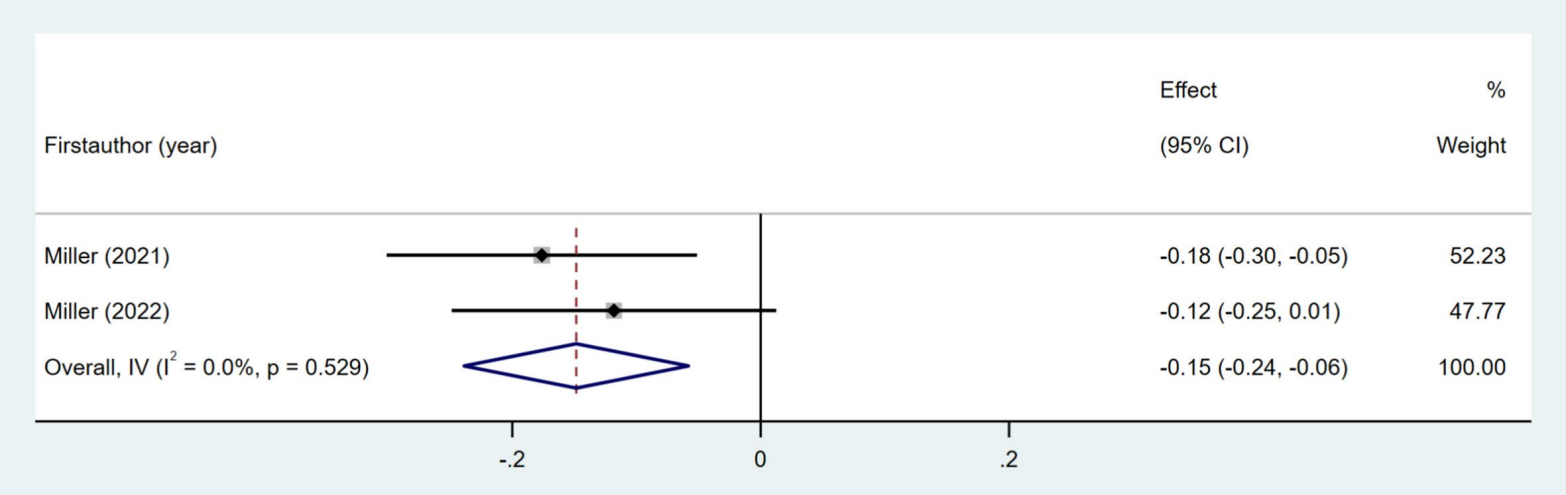
Forest plot for the relationship between area deprivation index and daily steps.

Two studies examined the association between the area deprivation index (ADI) and daily step count (31, 32). As shown in Figure 19, the meta-analysis indicated a weak negative correlation (Fisher’s *z* = −0.15; 95% CI, −0.24 to −0.06; meta *r* = −0.15; 95% CI, −0.24 to −0.06).

### 3.6 Sensitivity analysis

Sensitivity analyses were conducted after excluding the walking performance data from each of the 27 studies, and the pooled effect values did not change significantly, suggesting that the results were relatively stable (Supplementary Figure 1). In addition, sensitivity analysis was conducted for the 16 contextual factors, which showed that the results changed in terms of age, area deprivation index, cardiorespiratory fitness, and balance. These findings suggested that the results of the meta-analysis for these four contextual factors were not stable (Supplementary Figure 2). The pooled effect sizes for other contextual factors exhibited no significant changes, suggesting that the results were relatively stable (Supplementary Figure 2).

### 3.7 Publication bias

Egger’s test was employed to measure publication bias regarding walking performance (number of steps per day) and a funnel plot was drawn. The funnel plot showed that the distribution of study sites was asymmetrical (Supplementary Figure 3). Egger’s test (*t* = 3.85, *p* = 0.001) also suggested a significant publication bias. Of the 16 contextual factors extracted, only walking speed was included in ≥10 studies, which was tested in terms of publication bias. The funnel plot is shown in Supplementary Figure 4, and Egger’s test (*t* = 0.58 *p* = 0.580) also suggested no significant publication bias.

## 4 Discussion

In this systematic review and meta-analysis, we found that the metrics used to describe the walking performance in patients with stroke include the daily step count, time in stepping, walking distance and walking bouts. Among these, daily step count emerged as the most frequently reported outcome measure. The meta-analysis showed that the average number of steps per day was 4,269 among individuals with stroke. We also identified nine key contextual factors associated with daily step count in this population: age, gait speed, walking endurance, quality of life, area deprivation index, self-efficacy, cardiorespiratory fitness, balance, and RMA.

Walking performance is poor in people with stroke. Stratified by time since stroke, patients with stroke showed mean daily step counts of 3,217 steps within the first 3 months, 4,729 steps between 3 to 6 months, and 4,196 steps more than 6 months. All of these values were below the recommended threshold of 6,500 steps per day (52). Walking can offer significant health benefits. Daily steps of more than 2,200 were found to be associated with lower mortality (53). Previous studies have indicated that approximately 6,000 steps/day may be an initial target for reducing vascular events in patients with mild ischemic stroke (54). However, the present meta-analysis revealed that patients with stroke did not achieve this target. Therefore, we recommend targeted interventions to increase daily step counts in inactive patients with stroke, which may reduce recurrent vascular events.

Understanding contextual factors associated with the daily step counts of patients with stroke may help guide walking intervention. This is the first systematic review and meta-analysis conducted to synthesize the contextual factors associated with walking performance after stroke. In this study, the contextual factors associated with the walking performance of patients with stroke were categorized into three dimensions using the contextual factors classification model as a guide: user context, environmental context, and task context. We categorized user contexts into demographic information, disease-related information, movement capacity, psychological status, and health status. In terms of demographic information, age was significantly associated with daily steps taken by patients with stroke, with fewer daily steps taken by older patients. Similarly, studies in stroke cohorts have found a significant association between age and physical activity (14). However, the results of sensitivity analyses showed that the correlation between age and daily steps was not significant after excluding the study conducted by Miller et al. (32). This finding suggests that the result for age as a contextual factor for the walking performance of individuals with stroke was not robust and needs further validation. Disease-related information, including NIHSS score, time since stroke, and post-stroke fatigue, showed no statistically significant correlation with post-stroke daily steps in the meta-analysis. This may be attributed to small sample sizes in original studies and inter-study heterogeneity. Future large-sample and high-quality studies are needed to further clarify this association.

Regarding the movement capacity dimension, gait speed, walking endurance, balance, and RMA were significantly and positively correlated with daily step counts, and walking endurance exhibited the strongest correlation. Gait dysfunction is a common sequela following stroke (55). Patients present with gait asymmetry (55), reduced walking speed (56), and increased energy expenditure during walking (57), which contribute to diminished walking endurance and, ultimately, reduced daily activity levels. Impaired balance elevates fall risk and diminishes confidence in ambulatory activities, thereby further reducing daily step count. The RMA consists of 13 items that measure the functional mobility of patients with stroke. The RMA was significantly correlated with daily steps, likely attributed to its comprehensive assessment, which included stair climbing, walking outside, and walking over uneven surfaces. These are widely believed to be important aspects of usual walking performance (58). Thus, assessing movement capacity holds significant value for predicting future daily step counts in stroke patients, a conclusion supported by several recent predictive studies (59–61).

However, possessing excellent movement capacity does not necessarily lead to superior walking performance. Previous study revealed that walking ability contributes to merely 35.9% of the variability in daily step count (28). In the psychological status dimension, we found that self-efficacy was significantly associated with daily steps. Self-efficacy refers to an individual’s confidence and subjective judgment regarding their ability to perform position transitions and walking activities across diverse environmental conditions (28, 37). Patients with stroke who have higher self-efficacy exhibit greater daily step counts. It is recommended that healthcare professionals effectively help enhance the self-efficacy of individuals with stroke in walking activities by breaking down walking training goals to accumulate incremental successes, integrating vicarious demonstrations of rehabilitation cases of similar situations, offering professional and specific rehabilitation feedback, and supporting the regulation of emotional and physiological states.

In addition, the results of the present study showed that the correlation between depression and daily steps was not significant, and heterogeneity was high. Subgroup analyses revealed that the different scales measuring depressive status were the source of heterogeneity, suggesting that the relationship between depression and step counts needs further exploration on the basis of standardized assessment methods.

In the health status dimension, cardiorespiratory fitness and quality of life both exhibited significant correlations with daily step count. Cardiorespiratory fitness is one of the most important determinants of cardiovascular disease, and it may significantly decrease after stroke. Patients with stroke who have impaired cardiorespiratory fitness are more prone to fatigue, which leads to reduced walking endurance and fewer daily steps (62). The quality of life serves as an indicator of patients’ physical and mental well-being. Our results indicated that there exists a moderate positive correlation between quality of life and daily step count. Therefore, early identification of patients with stroke who have impaired cardiorespiratory fitness and reduced quality of life may facilitate the recognition of individuals with poor walking performance.

In the environmental context, factors associated with walking performance in patients with stroke included weather conditions and community environmental. However, due to heterogeneity across studies, only area deprivation index (ADI) within community environmental were included in the meta-analysis. The ADI is a composite index of neighborhood disadvantage that includes various indicators of housing quality and crowding, poverty, education, and employment (32). Our results showed a significant relationship between ADI and real-world walking activity, where higher ADI was associated with fewer real-world walking steps. Therefore, we recommend that policy and guideline makers establish localized ADI assessment frameworks, prioritize patients with stroke in high-deprivation areas, and mitigate the socioeconomic disparities among patients with stroke across different regions through targeted measures, including enhancing the accessibility of primary healthcare resources and improving housing and community environments. Moreover, sensitivity analyses suggested that the results of ADI were unstable, and further studies are needed in this regard. Additionally, while sufficient studies are lacking to conduct a meta-analysis of other environmental factors potentially associated with walking performance in patients with stroke, their impact remains non-negligible.

This study summarized task context as movement behaviors other than walking activities. People have a limited amount of time in the day; thus, changes in walking performance are accompanied by changes in the timing of other activities. So far, some studies have explored the associations and variations between 24-h movement behaviors (5, 63), but the evidence from these studies cannot be quantitatively synthesized. More studies in this area should be conducted to gain a deeper insight into task contexts related to walking performance after stroke.

However, this study may be affected by the following limitations: (1) We only extracted walking performance data from the literature that included both walking performance and associated factors. This strategy could compromise the completeness of qualitative and quantitative assessment of the current state of walking performance. Additionally, the meta-analyzed results for daily step counts exhibited high heterogeneity. Despite efforts to identify potential sources through subgroup analyses and meta-regression, no definitive explanatory factors were found. As a result, the generalizability of these findings warrants cautious interpretation. (2) Only observational studies were included in our meta-analysis; therefore, the factors reported by interventional studies may have been ignored. (3) Due to challenges in combining different effect sizes across studies, correlation coefficients were selected as effect sizes to pool data, increasing the risk of reverse causation. (4) Our study did not include grey literature, potentially resulting in incomplete evidence synthesis and increasing the risk of publication bias.

## 5 Conclusion

This systematic review and meta-analysis summarized the assessment indicators and contextual factors for walking performance among people had a stroke. These findings may guide the design of context-aware interventions, but the causal relationship between contextual factors and step counts needs further studies.
