# Supplementary figures and images for: Contextual factors associated with walking performance after stroke: a systematic review and meta-analysis
Source: Front Neurol. 2025 Sep 24;16:1635024. doi: 10.3389/fneur.2025.1635024 (PMC12504098; doi:10.3389/fneur.2025.1635024)

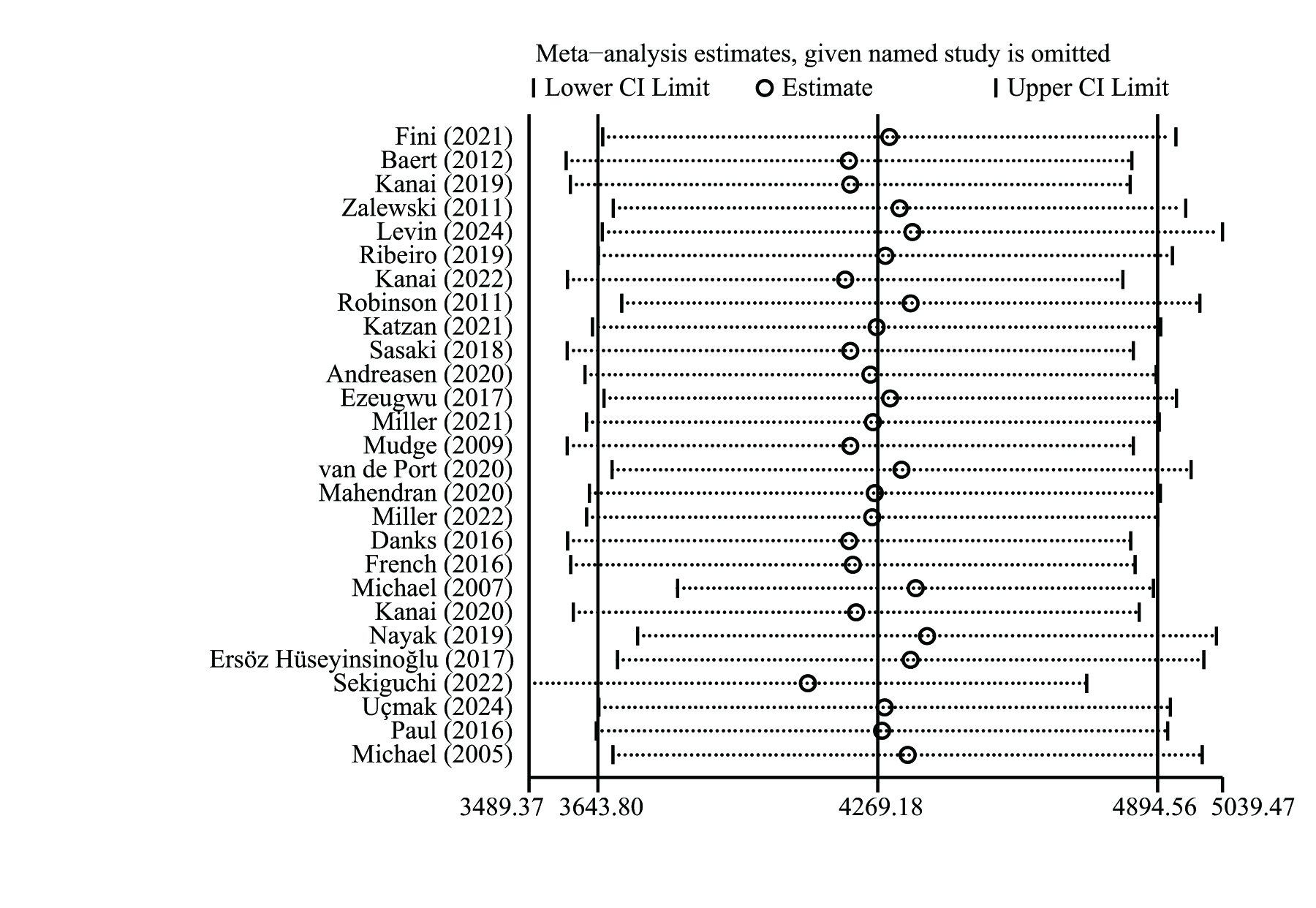


Supplemental Fig 1. Sensitivity analysis of daily steps in patients with stroke.

Supplement: Supplementary file 1 [file Supplementary_file_1.docx]

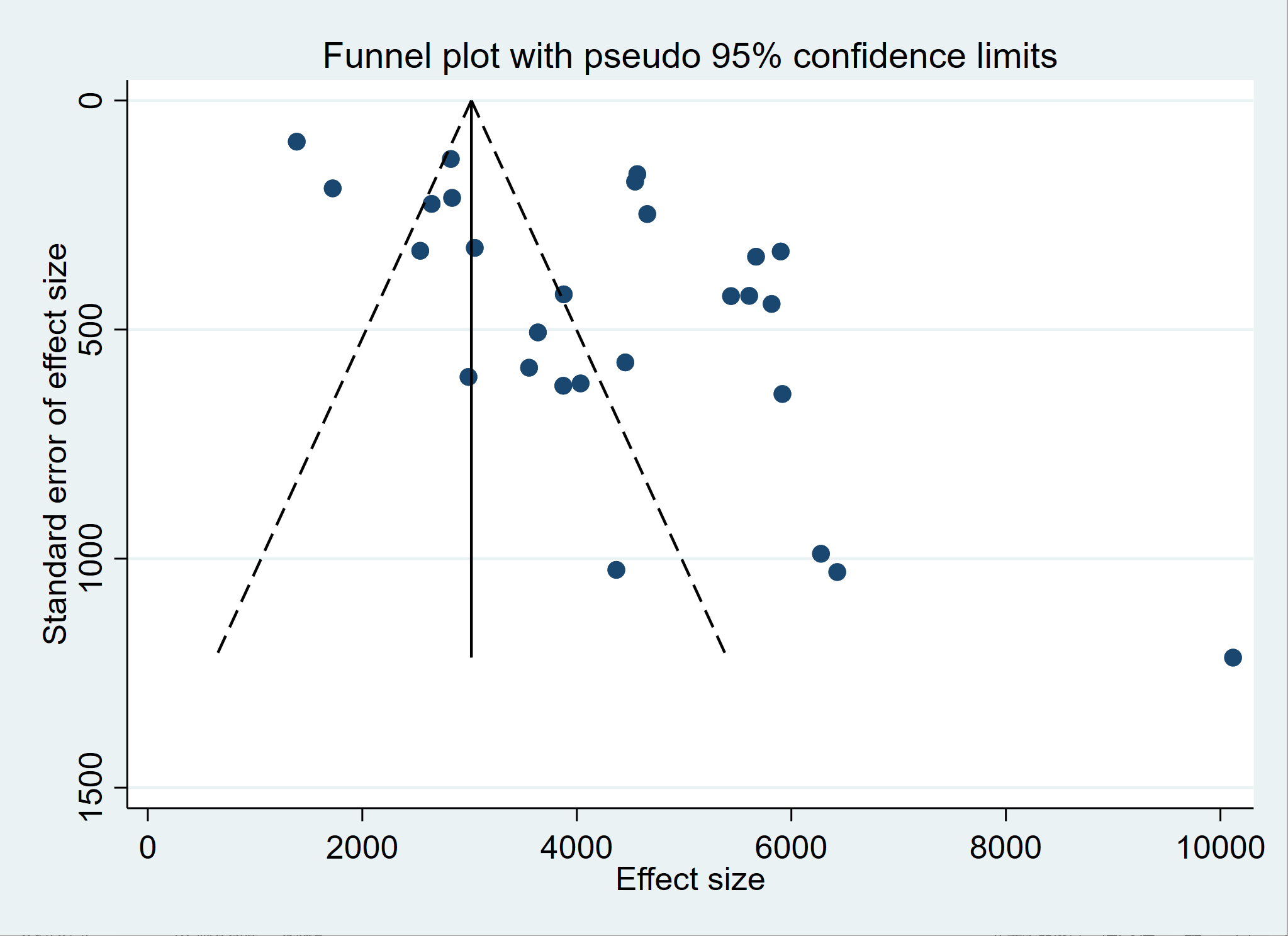


Supplemental Fig 3. Funnel plot of the included studies (Daliy steps).

Supplement: Supplementary file 3 [file Supplementary_file_3.docx]

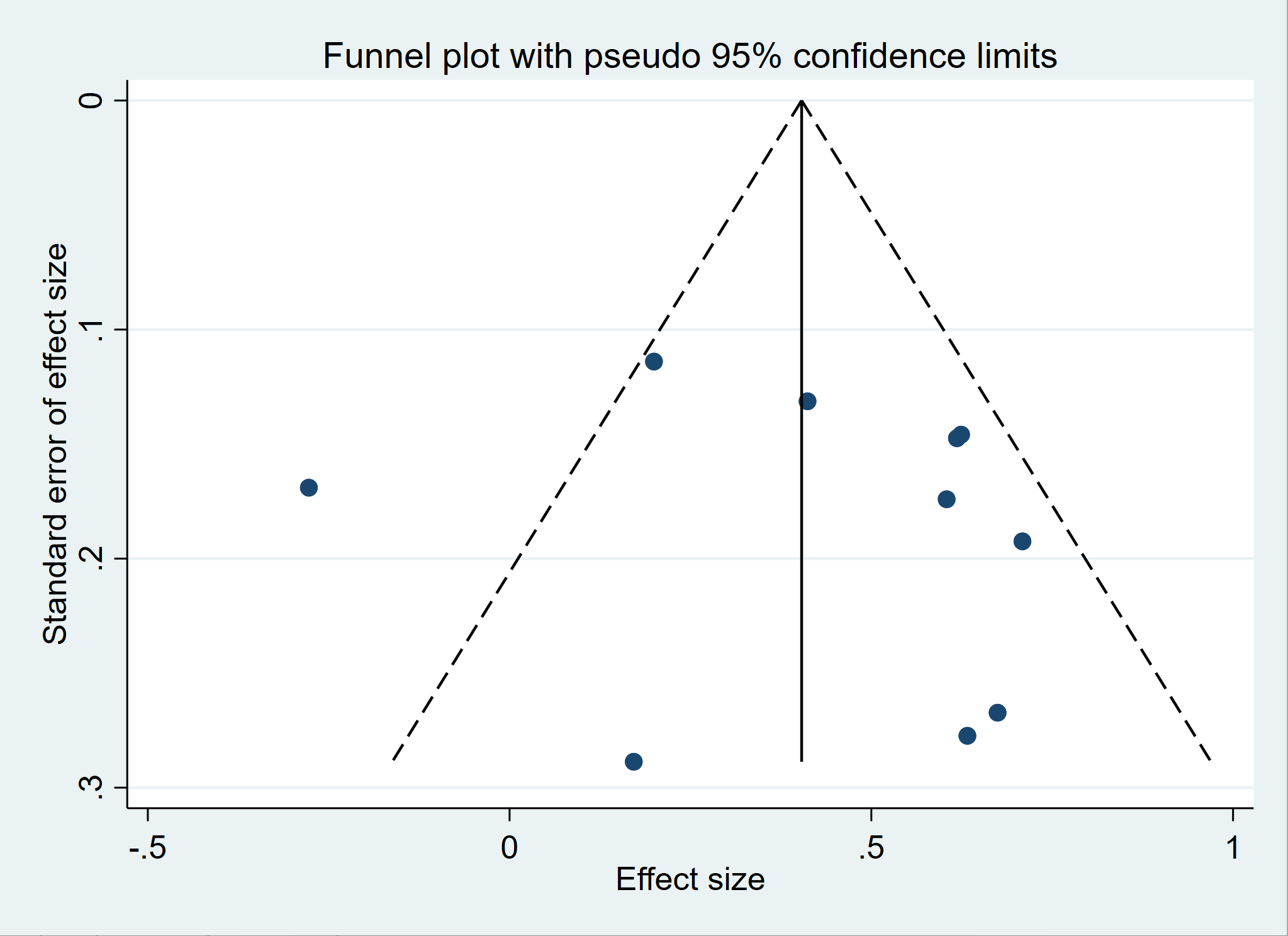


Supplemental Fig 4. Funnel plot of the included studies (Gait speed).

Supplement: Supplementary file 4 [file Supplementary_file_4.docx]
