# Supplementary material for: Contextual factors associated with walking performance after stroke: a systematic review and meta-analysis
Source: Front Neurol. 2025 Sep 24;16:1635024. doi: 10.3389/fneur.2025.1635024 (PMC12504098; doi:10.3389/fneur.2025.1635024)
Supplement: Supplementary file 2 [file Supplementary_file_2.docx]

Supplemental Fig 2. Sensitivity analysis of contextual factors with daily steps in patients with stroke.

(a) Age; (b) Gender; (c) Time since stroke; (d) Fatigue; (e) Cognitive status; (f) Quality of life; (g) Gait speed; (h) Walking endurance; (i) Area deprivation index; (j) Depression; (k) Cardiorespiratory fitness; (l) Balance ability (m) NHISS; (n) Economy of gait; (o) Self-efficacy; and (p) Rivermead motor assessment.
