# Supplementary material for: Contextual factors associated with walking performance after stroke: a systematic review and meta-analysis
Source: Front Neurol. 2025 Sep 24;16:1635024. doi: 10.3389/fneur.2025.1635024 (PMC12504098; doi:10.3389/fneur.2025.1635024)
Supplement: Supplementary file 5 [file Table_1.docx]

To ensure the comprehensiveness of the literature search results, the search keywords were expanded to movement behavior, including physical activity and sedentary behavior. The search strategies were shown in Table S1.

Table S1. Search strategy

|  |  | Search strategy |
| --- | --- | --- |
| PubMed | #1 | ((((((((((((((((("self report"[MeSH Terms]) OR ("acceleromet*"[Title/Abstract])) OR ("actigraph*"[Title/Abstract])) OR ("actiwatch*"[Title/Abstract])) OR ("motion logger*"[Title/Abstract])) OR ("motionlogger*"[Title/Abstract])) OR ("pedometer*"[Title/Abstract])) OR ("step*"[Title/Abstract])) OR ("self report"[Title/Abstract])) OR ("self-report"[Title/Abstract])) OR ("armband"[Title/Abstract])) OR ("activity tracker"[Title/Abstract])) OR ("Activity monitor"[Title/Abstract])) OR (Stepwatch[Title/Abstract])) OR ("Behavioural mapping"[Title/Abstract])) OR (Sensewear[Title/Abstract])) OR ("Sense-wear"[Title/Abstract])) OR (Questionnaire[Title/Abstract]) |
|  | #2 | (((((("stroke"[MeSH Terms]) OR ("cerebrovascular accident"[Title/Abstract])) OR ("brain vascular accident"[Title/Abstract])) OR ("hemorrhagic stroke"[Title/Abstract])) OR ("ischemic stroke"[Title/Abstract])) OR ("cerebral infarction"[Title/Abstract])) OR (stroke[Title/Abstract]) |
|  | #3 | (((((((Exercise[MeSH Terms]) OR ("physical activity"[Title/Abstract])) OR ("physical inactivity"[Title/Abstract])) OR ("sedentary behavior"[Title/Abstract])) OR ("sedentary time"[Title/Abstract])) OR ("movement behavior"[Title/Abstract])) OR ("sedentary lifestyle"[Title/Abstract])) OR (Exercise[Title/Abstract]) |
|  | #4 | ((((((((((((((((((((("risk factors"[MeSH Terms]) ) OR ("influence factor*"[Title/Abstract])) OR ("risk factors"[Title/Abstract])) OR ("influencing factor*"[Title/Abstract])) OR ("impact factor*"[Title/Abstract])) OR ("relevant factor*"[Title/Abstract])) OR ("relative factor*"[Title/Abstract])) OR ("correlative factor*"[Title/Abstract])) OR ("associated factor*"[Title/Abstract])) OR ("predictive factor*"[Title/Abstract])) OR ("environment*"[Title/Abstract])) OR ("social environment"[Title/Abstract])) OR ("physical environment"[Title/Abstract])) OR (weather[Title/Abstract])) OR (location[Title/Abstract])) OR (time[Title/Abstract])) OR (week*[Title/Abstract])) OR (context[Title/Abstract])) OR (temperture[Title/Abstract])) OR ("correlate*"[Title/Abstract])) OR ("risk factors"[Title/Abstract]) |
|  | #5 | "randomized controlled trial"[Title/Abstract] |
|  | #6 | English[Language] |
|  | #7 | #1 and#2 and#3and#4 |
|  | #8 | #7 not#5 |
|  | #9 | #8 and#6 |
| Web of science | #1 | (TI=(Exercise OR “physical activity” OR “physical inactivity” OR “sedentary behavior” OR “sedentary time” OR “sedentary lifestyle” OR “movement behavior”)) OR AB=(Exercise OR “physical activity” OR “physical inactivity” OR “sedentary behavior” OR “sedentary time” OR “sedentary lifestyle” OR “movement behavior”) |
|  | #2 | (TI=(“stroke” OR “cerebrovascular accident” OR “brain vascular accident” OR “hemorrhagic stroke” OR “ischemic stroke” OR “cerebral infarction”)) OR AB=(“stroke” OR “cerebrovascular accident” OR “brain vascular accident” OR “hemorrhagic stroke” OR “ischemic stroke” OR “cerebral infarction”) |
|  | #3 | (((((((((((((((((TI=("acceleromet*")) OR TI=("actigraph*")) OR TI=( "actiwatch*")) OR TI=(“motion logger*”)) OR TI=(“motionlogger*”)) OR TI=(“pedometer*”)) OR TI=( “step*”)) OR TI=(“self report”)) OR TI=(“self-report”)) OR TI=(“armband”)) OR TI=(“activity tracker”)) OR TI=(“Activity monitor” )) OR TI=(Stepwatch)) OR TI=(“Behavioural mapping”)) OR TI=(Sensewear)) OR TI=(“Sense-wear”)) OR TI=(Questionnaire)) OR AB=("acceleromet*" OR "actigraph*" OR "actiwatch*" OR “motion logger*” OR “motionlogger*” OR “pedometer*” OR “step*” OR “self report” OR “self-report” OR “armband” OR “activity tracker” OR “Activity monitor” OR Stepwatch OR “Behavioural mapping” OR Sensewear OR “Sense-wear” OR Questionnaire) |
|  | #4 | (TI=(“risk factors” OR “influence factor*” OR “influencing factor*” OR “impact factor*” OR “relevant factor*” OR “relative factor*” OR “correlative factor*” OR “associated factor*” OR “predictive factor*” OR “environment*” OR “social environment” OR “physical environment” OR weather OR location OR time OR week* OR context OR temperture OR “correlate*”)) OR AB=(“risk factors” OR “influence factor*” OR “influencing factor*” OR “impact factor*” OR “relevant factor*” OR “relative factor*” OR “correlative factor*” OR “associated factor*” OR “predictive factor*” OR “environment*” OR “social environment” OR “physical environment” OR weather OR location OR time OR week* OR context OR temperture OR “correlate*”) |
|  | #5 | #4 AND #3 AND #2 AND #1 |
|  | #6 | TI=("randomized controlled trial") |
|  | #7 | (#5) NOT #6 |
|  | #8 | #7 and English (Languages) |
| Embase | #1 | ('pedometer'/exp OR 'pedometer') AND [embase]/lim |
|  | #2 | ('exercise'/exp OR 'exercise' OR 'physical activity'/exp OR 'physical activity' OR 'sedentary lifestyle'/exp OR 'sedentary lifestyle') AND [embase]/lim |
|  | #3 | ('activity, physical':ab,ti OR 'physical activity':ab,ti OR 'exercise performance':ab,ti OR 'exertion':ab,ti OR 'physical effort':ab,ti OR 'physical exercise':ab,ti OR 'physical exertion':ab,ti OR 'physical work-out':ab,ti OR 'physical workout':ab,ti OR 'exercise':ab,ti OR 'sedentary behavior':ab,ti OR 'sedentary behaviour':ab,ti OR 'sedentary life style':ab,ti OR 'sedentary lifestyle':ab,ti) AND [embase]/lim |
|  | #4 | (('relative risk':ab,ti OR 'risk factors':ab,ti OR 'risk factor':ab,ti OR 'environment determinant':ab,ti OR 'environment':ab,ti OR 'weather change':ab,ti OR 'weather'/exp OR 'weather') AND ('risk factor'/exp OR 'risk factor') OR 'environment, social':ab,ti OR 'interpersonal climate':ab,ti OR 'social climate':ab,ti OR 'social context':ab,ti OR 'social environment':ab,ti OR 'weather':ab,ti OR 'location':ab,ti OR 'time':ab,ti OR 'week*':ab,ti OR 'context':ab,ti OR 'temperture':ab,ti) AND [embase]/lim |
|  | #5 | ('risk factor'/exp OR 'risk factor' OR 'weather'/exp OR 'weather' OR 'social environment'/exp OR 'social environment' OR 'environment'/exp OR 'environment') AND [embase]/lim |
|  | #6 | ('lifecorder ex':ab,ti OR 'sw-651 (pedometer)':ab,ti OR 'sw-700 (pedometer)':ab,ti OR 'walk4life':ab,ti OR 'acceleromet*':ab,ti OR 'actigraph*':ab,ti OR 'actiwatch*' OR 'motion logger*':ab,ti OR 'motionlogger*':ab,ti OR 'pedometer*':ab,ti OR 'step*':ab,ti OR 'self report':ab,ti OR 'self-report':ab,ti OR 'armband':ab,ti OR 'activity tracker':ab,ti OR 'activity monitor':ab,ti OR 'stepwatch':ab,ti OR 'behavioural mapping':ab,ti OR 'sensewear':ab,ti OR 'sense-wear':ab,ti OR 'questionnaire':ab,ti) AND [embase]/lim |
|  | #7 | ('cerebrovascular accident'/exp OR 'cerebrovascular accident') AND [embase]/lim |
|  | #8 | ('accident, cerebrovascular':ab,ti OR 'acute stroke':ab,ti OR 'apoplectic stroke':ab,ti OR 'apoplexia':ab,ti OR 'apoplexy':ab,ti OR 'brain accident':ab,ti OR 'brain attack':ab,ti OR 'brain vascular accident' OR 'cerebral apoplexia':ab,ti OR 'cerebral stroke':ab,ti OR 'cerebral vascular accident':ab,ti OR 'cerebro vascular accident':ab,ti OR 'cerebrum vascular accident':ab,ti OR 'cva':ab,ti OR 'stroke':ab,ti OR 'thrombotic stroke':ab,ti OR 'cerebrovascular accident':ab,ti) AND [embase]/lim |
|  | #9 | #7 OR #8 |
|  | #10 | #1 OR #6 |
|  | #11 | #2 OR #3 |
|  | #12 | #4 OR #5 |
|  | #13 | #9 AND #10 AND #11 AND #12 |
|  | #14 | ('randomized controlled trial':it OR 'randomized controlled trial':ti,ab,kw) AND [embase]/lim |
|  | #15 | #13 NOT #14 AND [embase]/lim |
| The Cochrnae Library | #1 | (“cerebrovascular accident”):ti,ab,kw OR (“brain vascular accident” ):ti,ab,kw OR (“hemorrhagic stroke”):ti,ab,kw OR (“ischemic stroke” ):ti,ab,kw OR (“cerebral infarction” ):ti,ab,kw |
|  | #2 | MeSH descriptor: [Stroke] explode all trees |
|  | #3 | MeSH descriptor: [Social Environment] explode all trees |
|  | #4 | MeSH descriptor: [Risk Factors] explode all trees |
|  | #5 | MeSH descriptor: [Weather] explode all trees |
|  | #6 | MeSH descriptor: [Exercise] explode all trees |
|  | #7 | MeSH descriptor: [Sedentary Behavior] explode all trees |
|  | #8 | #1 OR #2 |
|  | #9 | (risk NEXT factor* ):ti,ab,kw OR (influence NEXT factor* ):ti,ab,kw OR (influencing NEXT factor* ):ti,ab,kw OR (impact NEXT factor*):ti,ab,kw OR (relevant NEXT factor* ):ti,ab,kw OR (relative NEXT factor* ):ti,ab,kw OR( correlative NEXT factor* ):ti,ab,kw OR (associated NEXT factor* ):ti,ab,kw OR (predictive NEXT factor* ):ti,ab,kw OR (environment* ):ti,ab,kw OR (“social environment” ):ti,ab,kw OR (“physical environment” ):ti,ab,kw OR (weather):ti,ab,kw OR (location):ti,ab,kw OR (time):ti,ab,kw OR (week*):ti,ab,kw OR (context):ti,ab,kw OR (device):ti,ab,kw OR (temperture):ti,ab,kw OR (correlate* ):ti,ab,kw |
|  | #10 | #3 OR #4 OR #5 OR #9 |
|  | #11 | (Exercise):ti,ab,kw OR (“physical activity” ):ti,ab,kw OR (“physical inactivity” ):ti,ab,kw OR (“sedentary behavior”):ti,ab,kw OR (“sedentary time” ):ti,ab,kw OR (“movement behavior” ):ti,ab,kw |
|  | #12 | #6 OR #7 OR #11 |
|  | #13 | #8 AND #10 AND #12 |
|  | #14 | ("randomized controlled trial"):ti,ab,kw (Word variations have been searched) |
|  | #15 | (acceleromet*):ti,ab,kw OR (actigraph*):ti,ab,kw OR (actiwatch*):ti,ab,kw OR (motion NEXT logger*):ti,ab,kw OR (motionlogger*):ti,ab,kw OR (pedometer*):ti,ab,kw OR (step*):ti,ab,kw OR (self NEXT report):ti,ab,kw OR (self-report):ti,ab,kw OR (armband):ti,ab,kw OR (activity NEXT tracker):ti,ab,kw OR (activity NEXT monitor):ti,ab,kw OR (Stepwatch):ti,ab,kw OR (“behavioural mapping”):ti,ab,kw OR (Sensewear):ti,ab,kw OR (Sense-wear):ti,ab,kw OR (Questionnaire):ti,ab,kw |
|  | #16 | #13 AND #15 |
|  | #17 | #16 NOT #14 |
