# Supplementary material for: Contextual factors associated with walking performance after stroke: a systematic review and meta-analysis
Source: Front Neurol. 2025 Sep 24;16:1635024. doi: 10.3389/fneur.2025.1635024 (PMC12504098; doi:10.3389/fneur.2025.1635024)
Supplement: Supplementary file 6 [file Table_2.docx]

Table S2 Characteristics of studies exploring contextual factors associated with post-stroke walking performance

| First Author | Year | Nation | Study Design | Measurement  Tool | Sample Size | Age | Sex(M/F) | Stroke Severity (NIHSS) | | Time Since Stroke | Walking Performance Metrics | Contextual Factors | Quality Score |
| --- | --- | --- | --- | --- | --- | --- | --- | --- | --- | --- | --- | --- | --- |
| Fini (1) | 2021 | Australia | Longitudinal | SWAB | 79 | 65 ±14 | 54/25 | 9 (4–13) ^*^ | | 153d (78–226d)^*^ | Steps/d: 3685 (638–6596) ^*^ | Gait speed, walking endurance, anxiety and depression, fatigue, cognition | 8 |
| Baert (2) | 2012 | Belgium | Cross-sectional | Yamax SW-200 pedometer | 16 | 61.9 ± 11.9 | 12/4 | 4.9 ± 4.4 | | NR | Steps/d: 6428 ± 4117 | Age, gender, RMA, gait speed, depression, cardiorespiratory fitness, quality of life, hours of daylight | 7 |
| Kanai (3) | 2019 | Japan | Cross-sectional | Fitbit One | 61 | 67.0 (55.0–74.0) ^*^ | 46/15 | 1.0 (0–1.0) ^*^ | | 4.0mo (3.5–5.2mo)^*^ | Steps/day: 5556.7 (3965.0–7471.3) ^*^ | Age, gender, NIHSS, gait speed, sidewalks, access to recreational facilities | 9 |
| Zalewski (4) | 2011 | USA | Cross-sectional | SAM | 17 | 71.3 ± 9.5 | 14/3 | NR | NR | | Steps/d: 2990 ± 2488 | Gait speed, walking endurance | 8 |
| Levin (5) | 2024 | Israel | Cross-sectional | Accelerometer | 37 | 59.0 (54.0–64.5) ^*^ | 23/14 | NR | ≤ 27mo, 41.7%;  ＞27mo, 58.3% | | steps/d: 2814.2 (1219.2–4517.2)^*^; Device users: 2609.0 (762.0–3050.5) ^*^; Independent walkers: 4185.0 (2586.0–8517.0) ^*^ | Gait speed, TUG, walking while talking test | 9 |
| Ribeiro (6) | 2019 | Brazil | Cross-sectional | SAM | 23 | 60 ± 9 | 14/9 | NR | 48 (26–68)^*^ | | Steps/d: 3878 ± 2029; percentage of steps taken at low (1–15 steps/min), medium (16–40 steps/min), and high cadences (> 40 steps/min) (without inactive): 76.93± 10.49, 20.92 ±9.54, 0.18 (0.0–4.35) ^*^; percentage of time spent in activity at low, medium, and high cadences: 48.80 ±17.78, 43.52± 15.23, 0.90 (0.0–17.03) ^*^ | Energy cost of walking | 8 |
| Kanai (7) | 2022 | Japan | Cross-sectional | Fitbit One | 80 | 65.9 ± 11.1 | 58/22 | 1.3 ± 0.5 | 4.9 ± 4.5mo | | Steps/day: 5900.6 ± 2947.3 | Age, gender, gait speed, walk score, NIHSS, working status, long-term care insurance | 7 |
| Robinson (8) | 2011 | USA | Cross-sectional | VKRFitness Twin Step Pedometer | 50 (44) † | 65.0 ± 8.4 | 27/23 | NR | 85.0 ± 89.9mo | | Steps/d：2540 ± 2176 | Age, no. of comorbidities, fatigue, depression, fall self-efficacy, balance self-efficacy, Importance (walk, transportation) | 7 |
| Luzum (9) | 2023 | Norway | Cross-sectional | ActivPAL | 453 | 72.49 ± 11.31 | 260/193 | < 3: 50.9%  3–5: 28,51%  6–10: 11.54%  >10: 9.05% | NR | | Waking time (min): LPA walking, 233.16 ±127.39; MPA walking, 251.66 ± 164.62; No. of walking bouts,1374.69 ± 691.61; Bout length (s/bout), 20.90 ±7.28 | Age, gender, cognition, education | 9 |
| Katzan (10) | 2021 | USA | Longitudinal | Fitbit Charge HR | 15 | 54.4 ± 12.1 | 9/6 | 0: 40%  1: 33.3%  3: 20%  4: 6.7% | NR | | Steps/d: 4368 ± 3968; ranging from 1140 to 14610 | Walking endurance, balance, gait speed | 10 |
| Kunkel (11) | 2015 | UK | Longitudinal | ActivPAL | 74 (61\30\44\37) ^†^ | 76, range (44-95) | 39\35 | NR | 23d, range(2-83d) | | Time spent walking (%), Mean (median) min – max: T1, 2 (0.4) 0–14; T2,7 (3.5) 0–21; T3, 8 (7) 0–24; T4, 9 (6) 0–36 | Side of stroke, Barthel, FAC, BBS, anxiety and depression, RMI, Star Cancellation Test | 8 |
| Sasaki (12) | 2018 | Japan | Cross-sectional | Fitbit One | 22 | 69.5 ± 12.0 | 16/6 | 1.7 ± 1.5 | 6.3 ± 6.2mo | | Steps/d: 6276.3 ± 4640.7 | Quality of life | 5 |
| Andreasen (13) | 2020 | USA | Cross-sectional | Fitbit One or Zip | 142 | 63.25 ± 11.32 | 77/65 | NR | 51.53 ± 64.08mo | | Steps/d: 4656.57 ± 2952.86 | Season, presence of precipitation | 4 |
| Ezeugwu (14) | 2017 | Canada | Cross-sectional | ActivPAL3 Micro | 30 | 63.8 ± 12.3 | 17/13 | NR | 3.6 ± 1.1mo | | Steps/d: 2590 (1891-5995) ^*^; stepping time (minutes/day): 68.4; sporadic steps (20-39 steps/minute): 4.7 (2.8-7.3) ^*^; purposeful steps (40-59 steps/minute): 8.8 (6.1-15.4) ^*^, slow steps (60-79 steps/minute):13.0 (7.6-29.6) ^*^, medium steps (80-99 steps/minute):11.9 (5.8-27.4) ^*^; brisk steps (100-119 steps/minute):1.2 (0.2-5.6) ^*^; fastest steps (>120 steps/minute): 0.2 (0.1-0.5) ^*^ | Age, time since stroke, gait speed, cognition, quality of life, sleep duration, sedentary time, standing time, sit-to-stand transitions | 6 |
| Miller (15) | 2021 | USA | Cross-sectional | FitBit One or Zip | 249 | 62.98 ± 11.94 | 120/129 | NR | 47.47 ± 60.21mo | | Steps/d: 4543 ±2793 | Age, gender, living situation, working status, marital status, ADL, walk score | 7 |
| Mudge (16) | 2009 | New Zealand | Cross-sectional | SAM | 49 | 67.4 ± 12.5 | 29/20 | NR | 66 ± 61mo | | Steps/d: 4765^*^, range (1225–21273); Percentage of time with no steps: 83%^*^, range(53–96); number of steps at low rate (＜30 steps/min): 2334 ± 565; Number of steps at high rate (＞60 steps/min): 655^*^, range (0–10590); Highest step rate in 60 minutes (max 60) (steps/min): 18.7^*^, range (5–89); Highest step rate in 1 minute (max 1) (steps/min): 81.5±11.1; Peak activity index (steps/min): 58.7 ± 10.6 | Age, gait speed, walking Endurance, RMI, RMA | 6 |
| van de Port (17) | 2020 | Netherlands | Cross-sectional | Tri-axial accelerometer | 38 | 61.9 ± 11.4 | 19/19 | NR | 56.5mo (64.8mo)^*^ | | Steps/d: 3048.3 ± 1983.1;  walking bouts a day(n):123.3 ± 61.3, short (92.5%), long (7.5%); gait duration (min/day): 32.5 ± 18.2; step frequency (steps/min): 90.3 ± 13.8 | Gait speed, TUG, balance, fall self-efficacy, depression | 6 |
| Mahendran (18) | 2020 | Australia | Longitudinal | ActivPAL^TM^ | 36/31/29 | 71 ± 14 | 25/11 | NR | NR | | Steps/d: 4452± 3430 (T1), 4623 ±2735(T2), 4946± 3732(T3);Time in long bouts (min), ≥300steps：16.3 ±23.6 (T1), 13.9± 15.0(T2), 17.4 ±24.9 (T3); Time in high intensity (>80 steps/minute):13.5 ± 17.9 (T1), 9.9 ±13.6 (T2), 16.3 ± 24.4 (T3) | Age, fatigue, depression, executive function, gait speed, walking endurance, PASE, self-efficacy, quality of life | 7 |
| Miller (19) | 2022 | USA | Cross-sectional | FitBit One or Zip | 282 | 63.43 ± 12.63 | 146/136 | NR | 46.99 ± 61.5mo | | Steps/d: 4563.73 ± 2696.78 | ADL, balance self-efficacy, living situation | 7 |
| Danks (20) | 2016 | USA | Cross-sectional | SAM | 55 | 54 ± 11 | 33/22 | NR | 47mo, range (4-366mo) | | Steps/d: 5816 ± 3293 | FGA, fatigue, depression, MCIR, Walk 12, balance self-efficacy, energy cost of transport | 5 |
| French (21) | 2016 | USA | Cross-sectional | SAM | 59 | 59 ± 11.2 | NR | NR | 44.3 ± 63.1mo | | Steps/d：5607 ± 3275 | performance based, self-efficacy | 6 |
| Michael (22) | 2007 | USA | Cross-sectional | SAM | 79 | 65, range (45-84) | 42/37 | 3.57, range (0-16) | 10.3mo, range (6-166 mo) | | Steps/d: 1389 ± 798; Low intensity: 624± 289; Medium intensity: 640 ± 536; High intensity: 83 ± 168 | Economy of gait, cardiorespiratory fitness, fatigue | 6 |
| Kanai (23) | 2020 | Japan | Cross-sectional | Fitbit One | 50 | 68.0 (53.8-77.0)^*^ | 40/10 | 1.0 (1.0–2.0)^*^ | 4.2mo (1.7-6.4mo)^*^ | | Steps/d: 5472.9 (3445.2-7399.9)^*^ | Quality of life | 6 |
| Nayak (24) | 2019 | India | Cross-sectional | Actigraph GT3X | 33(30)^†^ | 58.4 ± 12.1 | 27/6 | NR |  | | Steps/d：1635 (1084.9, 2434.3)^*^ | Quality of life | 7 |
| Ersöz Hüseyinsinoğlu (25) | 2017 | Istanbul | Cross-sectional | OMRON step Counter | 85 | 64.7 ± 10.2 | 50/35 | NR | 56.63 ± 31.17d | | Steps/d: 2646.5 ± 1235.6; Walking distance:1.8 ± 1.0 | Age, BMI, education, time since stroke，apathy, cognition, depression | 6 |
| Kossi (26) | 2024 | Benin | Cross-sectional | SWAB | 21 | 56.24 ± 12.86 | 18/3 | NR | 12 ± 0.00mo | | Steps/d: 2767^*^ | Cardiorespiratory fitness | 6 |
| Sekiguchi (27) | 2022 | Japan | Cross-sectional | ActiGraph | 14 | 59.3 ± 8.9 | 11/3 | NR | NR | | Steps/d: 10118.0 ± 4550.2 | The difference in maximum knee extension in the stance phase and ankle plantarflexion at foot off on the non-PS | 6 |
| Uçmak (28) | 2024 | Istanbul | Cross-sectional | SenseWear Pro 3 Armband | 32 | 52.31 ± 14.76 | 19/13 | NR | NR | | Steps/d: 3873.44 ± 3521.78 | Kinesiophobia, fatigue, quality of life | 6 |
| Paul (29) | 2016 | UK | Cross-sectional | ActivPAL^TM^ | 22 (21)^†^ | 55.9 ± 9.9 | 10/12 | NR | 4.2 ± 4.0y | | Steps/d: 4035± 2830; walking time(h): walking time (＞20 steps/min): 1.0 ± 0.6; Incidental movement (1-19 steps/min): 2.7±1.2; Sporadic movement (20-39 steps/min): 0.2 ± 0.1; Purposeful steps(40-59 steps/min): 0.2 ± 0.1; Slow walking (60-79 steps/min): 0.2 ± 0.3; Medium walking(80-99 steps/min): 0.1 ± 0.3; Brisk walking(100 - 119steps/min): 0.03±0.31; Faster locomotion(＞120 steps/min): 0.01 ± 0.02 | Gait speed, fatigue | 5 |
| Michael (30) | 2005 | USA | Cross-sectional | SAM | 50 | 65, range (45-84) | 28/22 | 3.57, range (0-16) | 10.3mo, range (6-166mo) | | Steps/d: 2837±1503 | Balance, gait speed, economy of gait, fractional utilization, cardiorespiratory fitness | 7 |

Abbreviations: M, male; F, female; NIHSS, National Institutes Hospital Stroke Scale; SWAB, SenseWear Armband; SAM, StepWatch Activity Monitor; NR, not reported; RMI, Rivermead Mobility Index; RMA, Rivermead Motor Assessment; TUG, timed up and go test; FAC, Functional Ambulation Category; ADL, Area Deprivation Index; MCIR, Modified Cumulative Illness Rating; PASE, Physical activity scale for the elderly; FGA, Functional Gait Assessment

^*^ Data are presented as median values (interquartile range).

^†^ Values in parenthesis indicate the sample size used in the statistical analysis after removal of subjects with missing data.

References

1. Fini NA, Bernhardt J, Churilov L, Clark R, Holland AE. A 2-Year Longitudinal Study of Physical Activity and Cardiovascular Risk in Survivors of Stroke. *Physical therapy* (2021) 101:pzaa205. doi: 10.1093/ptj/pzaa205.

2. Baert I, Feys H, Daly D, Troosters T, Vanlandewijck Y. Are Patients 1 Year Post-Stroke Active Enough to Improve Their Physical Health? *Disabil Rehabil* (2012) 34:574-80. doi: 10.3109/09638288.2011.613513.

3. Kanai M, Izawa KP, Kubo H, Nozoe M, Mase K, Koohsari MJ, et al. Association of Perceived Built Environment Attributes with Objectively Measured Physical Activity in Community-Dwelling Ambulatory Patients with Stroke. *Int J Environ Res Public Health* (2019) 16:3908. doi: 10.3390/ijerph16203908

4. Zalewski KR, Dvorak L. Barriers to Physical Activity between Adults with Stroke and Their Care Partners. *Top Stroke Rehabil* (2011) 18 Suppl 1:666-75. doi: 10.1310/tsr18s01-666.

5. Levin C, Bachar-Kirshenboim Y, Rand D. Daily Steps, Walking Tests, and Functioning in Chronic Stroke; Comparing Independent Walkers to Device-Users. *Physiother Res Int* (2024) 29:e2035. doi: 10.1002/pri.2035.

6. Ribeiro JAM, Oliveira SG, Thommazo-Luporini LD, Monteiro CI, Phillips SA, Catai AM, et al. Energy Cost During the 6-Minute Walk Test and Its Relationship to Real-World Walking after Stroke: A correlational, Cross-Sectional Pilot study. *Phys Ther* (2019) 99:1656-66. doi: 10.1093/ptj/pzz122

7. Kanai M, Izawa KP, Kubo H, Nozoe M, Shimada S. Objectively Measured Physical Activity Was Not Associated with Neighborhood Walkability Attributes in Community-Dwelling Patients with Stroke. *Sci Rep* (2022) 12:3475. Epub 2022/03/05. doi: 10.1038/s41598-022-07467-y

8. Robinson CA, Shumway-Cook A, Ciol MA, Kartin D. Participation in Community Walking Following Stroke: Subjective Versus Objective Measures and the Impact of Personal Factors. *Physical therapy* (2011) 91:1865-76. doi: 10.2522/ptj.20100216

9. Luzum G, Gunnes M, Lydersen S, Saltvedt I, Tan X, Thingstad P, et al. Physical Activity Behavior and Its Association with Global Cognitive Function Three Months after Stroke: The nor-Coast Study†. *Physical therapy* (2023) 103(12). doi: 10.1093/ptj/pzad092

10. Katzan I, Schuster A, Kinzy T. Physical Activity Monitoring Using a Fitbit Device in Ischemic Stroke Patients: Prospective Cohort Feasibility Study. *JMIR mHealth and uHealth* (2021) 9:e14494. Epub 2021/01/20. doi: 10.2196/14494

11. Kunkel D, Fitton C, Burnett M, Ashburn A. Physical Inactivity Post-Stroke: A 3-Year Longitudinal Study. *Disabil Rehabil* (2015) 37:304-10. doi: 10.3109/09638288.2014.918190

12. Sasaki S, Kanai M, Shinoda T, Morita H, Shimada S, Izawa KP. Relation between Health Utility Score and Physical Activity in Community-Dwelling Ambulatory Patients with Stroke: A Preliminary Cross-Sectional Study. Topics in stroke rehabilitation (2018):1-5. Epub 2018/07/25. doi: 10.1080/10749357.2018.1492775

13. Andreasen SC, Wright TR, Crenshaw JR, Reisman DS, Knarr BA. Relationships of Linear and Non-Linear Measurements of Post-Stroke Walking Activity and Their Relationship to Weather. *Front Sports Act Living* (2020) 2:551542. doi: 10.3389/fspor.2020.551542

14. Ezeugwu VE, Manns PJ. Sleep Duration, Sedentary Behavior, Physical Activity, and Quality of Life after Inpatient Stroke Rehabilitation. *J Stroke Cerebrovasc Dis* (2017) 26:2004-12. doi: 10.1016/j.jstrokecerebrovasdis.2017.06.009

15. Miller A, Pohlig RT, Reisman DS. Social and Physical Environmental Factors in Daily Stepping Activity in Those with Chronic Stroke. *Topics in stroke rehabilitation* (2021) 28:161-9. Epub 2020/08/11. doi: 10.1080/10749357.2020.1803571

16. Mudge S, Stott NS. Timed Walking Tests Correlate with Daily Step Activity in Persons with Stroke. *Arch Phys Med Rehabil* (2009) 90:296-301. doi: 10.1016/j.apmr.2008.07.025.

17. van de Port I, Punt M, Meijer JW. Walking Activity and Its Determinants in Free-Living Ambulatory People in a Chronic Phase after Stroke: A Cross-Sectional Study. *Disabil Rehabil* (2020) 42:636-41. doi: 10.1080/09638288.2018.1504327

18. Mahendran N, Kuys SS, Brauer SG. Which Impairments, Activity Limitations and Personal Factors at Hospital Discharge Predict Walking Activity across the First 6 Months Poststroke? *Disabil Rehabil* (2020) 42:763-9. doi: 10.1080/09638288.2018.1508513

19. Miller A, Pohlig RT, Reisman DS. Relationships among Environmental Variables, Physical Capacity, Balance Self-Efficacy, and Real-World Walking Activity Post-Stroke. *Neurorehabil Neural Repair* (2022) 36:535-44. doi:10.1177/15459683221115409

20. Danks KA, Pohlig RT, Roos M, Wright TR, Reisman DS. Relationship between Walking Capacity, Biopsychosocial Factors, Self-Efficacy, and Walking Activity in Persons Poststroke. *J Neurol Phys Ther* (2016) 40:232-8. doi: 10.1097/NPT.0000000000000143

21. French MA, Moore MF, Pohlig R, Reisman D. Self-Efficacy Mediates the Relationship between Balance/Walking Performance, Activity, and Participation after Stroke. *Top Stroke Rehabil* (2016) 23:77-83. doi: 10.1080/10749357.2015.1110306

22. Michael K, Macko RF. Ambulatory Activity Intensity Profiles, Fitness, and Fatigue in Chronic Stroke. *Top Stroke Rehabil* (2007) 14:5-12. doi:10.1310/tsr1402-5

23. Kanai M, Izawa KP, Kubo H, Nozoe M, Mase K, Shimada S. Association of Health Utility Score with Physical Activity Outcomes in Stroke Survivors. *Int J Environ Res Public Health* (2020) 18:251. doi:10.3390/ijerph18010251

24. Nayak P, Kumaran SD, Babu AS, Maiya AG, Solomon JM. Levels of Physical Activity and Quality of Life among Community-Dwelling Adults with Stroke in a Developing Country. *European Journal of Physiotherapy* (2019) 23:165-70. doi: 10.1080/21679169.2019.1663927

25. Ersöz Hüseyinsinoğlu B, Kuran Aslan G, Tarakci D, Razak Özdinçler A, Küçükoğlu H, Baybaş S. Physical Activity Level of Ambulatory Stroke Patients: Is It Related to Neuropsychological Factors? *Noropsikiyatri Arsivi* (2017) 54:155-61. doi: 10.5152/npa.2016.12760

26. Kossi O, Bonnechère B, Agbetou M, Somasse R, Hokpo A, Houehanou YCN, et al. Relationships between Cardiorespiratory Fitness, Physical Activity Practices, and Functional Outcomes One-Year Post-Stroke in Northern Benin: A Case–Control Study. *Top Stroke Rehabil* (2024) 31:104-15. doi: 10.1080/10749357.2023.2207286

27. Sekiguchi Y, Honda K, Izumi SI. Effect of Walking Adaptability on an Uneven Surface by a Stepping Pattern on Walking Activity after Stroke. *Front Hum Neurosci* (2022) 15. doi: 10.3389/fnhum.2021.762223.

28. Uçmak GS, Kilinç M. The Effects of Kinesiophobia, Fatigue, and Quality of Life on Physical Activity in Patients with Stroke. *Top Stroke Rehabil* (2024) 31:788-94. doi: 10.1080/10749357.2024.2333159

29. Paul L, Brewster S, Wyke S, Gill JMR, Alexander G, Dybus A, et al. Physical Activity Profiles and Sedentary Behaviour in People Following Stroke: A Cross-Sectional Study. *Disabil Rehabil* (2016) 38:362-7. doi: 10.3109/09638288.2015.1041615

30. Michael KM, Allen JK, Macko RF. Reduced Ambulatory Activity after Stroke: The Role of Balance, Gait, and Cardiovascular Fitness. *Arch Phys Med Rehabil* (2005) 86:1552-6. doi: 10.1016/j.apmr.2004.12.026
