# Supplementary material for: Contextual factors associated with walking performance after stroke: a systematic review and meta-analysis
Source: Front Neurol. 2025 Sep 24;16:1635024. doi: 10.3389/fneur.2025.1635024 (PMC12504098; doi:10.3389/fneur.2025.1635024)
Supplement: Supplementary file 7 [file Table_3.docx]

Table S3 Risk of bias in included studies

| First author | Year | Define the source of information (survey, record review) | List inclusion and exclusion criteria for exposed and unexposed subjects (cases and controls) or refer to previous publications | Indicate time period used for identifying patients | Indicate whether or not subjects were consecutive if not population - based | Indicate if evaluators of subjective components of study were masked to other aspects of the status of the participants | Describe any assessments undertaken for quality assurance purposes (e.g., test/retest of primary outcome measurements) | Explain any patient exclusions from analysis | Describe how confounding was assessed and/or controlled | If applicable, explain how missing data were handled in the analysis | Summarize patient response rates and completeness of data collection | Clarify what follow - up, if any, was expected and the percentage of patients for which incomplete data or follow - up was obtained | Quality score | Quality level |
| --- | --- | --- | --- | --- | --- | --- | --- | --- | --- | --- | --- | --- | --- | --- |
| Fini (1) | 2021 | 1 | 1 | 1 | 1 | 1 | 1 | 1 | 0 | 0 | 0 | 1 | 8 | High |
| Baert (2) | 2012 | 1 | 1 | 1 | 1 | 1 | 1 | 1 | 0 | 0 | 1 | 0 | 8 | High |
| Kanai (3) | 2019 | 1 | 1 | 1 | 1 | 1 | 1 | 1 | 1 | 0 | 1 | 0 | 9 | High |
| Zalewski (4) | 2011 | 1 | 0 | 0 | 1 | 1 | 1 | 1 | 1 | 1 | 1 | 0 | 8 | High |
| Levin (5) | 2024 | 1 | 1 | 1 | 1 | 1 | 1 | 1 | 0 | 1 | 1 | 0 | 9 | High |
| Ribeiro (6) | 2019 | 1 | 1 | 1 | 1 | 1 | 0 | 1 | 0 | 1 | 1 | 0 | 8 | High |
| Kanai (7) | 2022 | 1 | 1 | 0 | 1 | 1 | 0 | 1 | 1 | 0 | 1 | 0 | 7 | Moderate |
| Robinson (8) | 2011 | 1 | 1 | 1 | 1 | 1 | 0 | 1 | 0 | 0 | 1 | 0 | 7 | Moderate |
| Luzum (9) | 2023 | 1 | 1 | 1 | 1 | 1 | 1 | 1 | 1 | 0 | 1 | 0 | 9 | High |
| Katzan (10) | 2021 | 1 | 1 | 1 | 1 | 1 | 1 | 1 | 1 | 0 | 1 | 1 | 10 | High |
| Kunkel (11) | 2015 | 1 | 1 | 1 | 1 | 1 | 0 | 1 | 0 | 0 | 1 | 1 | 8 | High |
| Sasaki (12) | 2018 | 1 | 1 | 0 | 1 | 1 | 0 | 1 | 0 | 0 | 0 | 0 | 5 | Moderate |
| Andreasen (13) | 2020 | 1 | 1 | 0 | 1 | 1 | 1 | 1 | 0 | 1 | 1 | 0 | 8 | High |
| Ezeugwu (14) | 2017 | 1 | 1 | 1 | 1 | 1 | 0 | 1 | 0 | 0 | 0 | 0 | 6 | Moderate |
| Miller (15) | 2021 | 1 | 1 | 1 | 1 | 1 | 1 | 0 | 1 | 0 | 0 | 0 | 7 | Moderate |
| Mudge (16) | 2009 | 1 | 1 | 1 | 0 | 1 | 0 | 1 | 1 | 0 | 0 | 0 | 6 | Moderate |
| van de Port (17) | 2020 | 1 | 1 | 1 | 0 | 1 | 1 | 1 | 0 | 0 | 0 | 0 | 6 | Moderate |
| Mahendran (18) | 2020 | 1 | 1 | 1 | 1 | 1 | 0 | 1 | 0 | 0 | 0 | 1 | 7 | Moderate |
| Miller (19) | 2022 | 1 | 1 | 1 | 1 | 1 | 1 | 0 | 1 | 0 | 0 | 0 | 7 | Moderate |
| Danks (20) | 2016 | 1 | 1 | 1 | 0 | 1 | 1 | 0 | 0 | 0 | 0 | 0 | 5 | Moderate |
| French (21) | 2016 | 1 | 1 | 1 | 1 | 1 | 1 | 0 | 0 | 0 | 0 | 0 | 6 | Moderate |
| Michael (22) | 2007 | 1 | 1 | 1 | 1 | 1 | 0 | 1 | 0 | 0 | 0 | 0 | 6 | Moderate |
| Kanai (23) | 2020 | 1 | 1 | 0 | 1 | 1 | 0 | 1 | 1 | 0 | 0 | 0 | 6 | Moderate |
| Nayak (24) | 2019 | 1 | 1 | 1 | 1 | 1 | 1 | 1 | 0 | 0 | 0 | 0 | 7 | Moderate |
| Ersöz Hüseyinsinoğlu (25) | 2017 | 1 | 1 | 1 | 1 | 1 | 1 | 0 | 0 | 0 | 0 | 0 | 6 | Moderate |
| Kossi (26) | 2024 | 1 | 1 | 1 | 1 | 1 | 0 | 1 | 0 | 0 | 0 | 0 | 6 | Moderate |
| Sekiguchi (27) | 2022 | 1 | 1 | 1 | 0 | 1 | 1 | 0 | 0 | 0 | 1 | 0 | 6 | Moderate |
| Uçmak (28) | 2024 | 1 | 1 | 1 | 1 | 1 | 1 | 0 | 0 | 0 | 0 | 0 | 6 | Moderate |
| Paul (29) | 2016 | 1 | 1 | 0 | 1 | 1 | 1 | 0 | 0 | 0 | 0 | 0 | 5 | Moderate |
| Michael (30) | 2005 | 1 | 1 | 1 | 1 | 1 | 1 | 1 | 0 | 0 | 0 | 0 | 7 | Moderate |

References

1. Fini NA, Bernhardt J, Churilov L, Clark R, Holland AE. A 2-Year Longitudinal Study of Physical Activity and Cardiovascular Risk in Survivors of Stroke. *Physical therapy* (2021) 101:pzaa205. doi: 10.1093/ptj/pzaa205.

2. Baert I, Feys H, Daly D, Troosters T, Vanlandewijck Y. Are Patients 1 Year Post-Stroke Active Enough to Improve Their Physical Health? *Disabil Rehabil* (2012) 34:574-80. doi: 10.3109/09638288.2011.613513.

3. Kanai M, Izawa KP, Kubo H, Nozoe M, Mase K, Koohsari MJ, et al. Association of Perceived Built Environment Attributes with Objectively Measured Physical Activity in Community-Dwelling Ambulatory Patients with Stroke. *Int J Environ Res Public Health* (2019) 16:3908. doi: 10.3390/ijerph16203908

4. Zalewski KR, Dvorak L. Barriers to Physical Activity between Adults with Stroke and Their Care Partners. *Top Stroke Rehabil* (2011) 18 Suppl 1:666-75. doi: 10.1310/tsr18s01-666.

5. Levin C, Bachar-Kirshenboim Y, Rand D. Daily Steps, Walking Tests, and Functioning in Chronic Stroke; Comparing Independent Walkers to Device-Users. *Physiother Res Int* (2024) 29:e2035. doi: 10.1002/pri.2035.

6. Ribeiro JAM, Oliveira SG, Thommazo-Luporini LD, Monteiro CI, Phillips SA, Catai AM, et al. Energy Cost During the 6-Minute Walk Test and Its Relationship to Real-World Walking after Stroke: A correlational, Cross-Sectional Pilot study. *Phys Ther* (2019) 99:1656-66. doi: 10.1093/ptj/pzz122

7. Kanai M, Izawa KP, Kubo H, Nozoe M, Shimada S. Objectively Measured Physical Activity Was Not Associated with Neighborhood Walkability Attributes in Community-Dwelling Patients with Stroke. *Sci Rep* (2022) 12:3475. Epub 2022/03/05. doi: 10.1038/s41598-022-07467-y

8. Robinson CA, Shumway-Cook A, Ciol MA, Kartin D. Participation in Community Walking Following Stroke: Subjective Versus Objective Measures and the Impact of Personal Factors. *Physical therapy* (2011) 91:1865-76. doi: 10.2522/ptj.20100216

9. Luzum G, Gunnes M, Lydersen S, Saltvedt I, Tan X, Thingstad P, et al. Physical Activity Behavior and Its Association with Global Cognitive Function Three Months after Stroke: The nor-Coast Study†. *Physical therapy* (2023) 103(12). doi: 10.1093/ptj/pzad092

10. Katzan I, Schuster A, Kinzy T. Physical Activity Monitoring Using a Fitbit Device in Ischemic Stroke Patients: Prospective Cohort Feasibility Study. *JMIR mHealth and uHealth* (2021) 9:e14494. Epub 2021/01/20. doi: 10.2196/14494

11. Kunkel D, Fitton C, Burnett M, Ashburn A. Physical Inactivity Post-Stroke: A 3-Year Longitudinal Study. *Disabil Rehabil* (2015) 37:304-10. doi: 10.3109/09638288.2014.918190

12. Sasaki S, Kanai M, Shinoda T, Morita H, Shimada S, Izawa KP. Relation between Health Utility Score and Physical Activity in Community-Dwelling Ambulatory Patients with Stroke: A Preliminary Cross-Sectional Study. Topics in stroke rehabilitation (2018):1-5. Epub 2018/07/25. doi: 10.1080/10749357.2018.1492775

13. Andreasen SC, Wright TR, Crenshaw JR, Reisman DS, Knarr BA. Relationships of Linear and Non-Linear Measurements of Post-Stroke Walking Activity and Their Relationship to Weather. *Front Sports Act Living* (2020) 2:551542. doi: 10.3389/fspor.2020.551542

14. Ezeugwu VE, Manns PJ. Sleep Duration, Sedentary Behavior, Physical Activity, and Quality of Life after Inpatient Stroke Rehabilitation. *J Stroke Cerebrovasc Dis* (2017) 26:2004-12. doi: 10.1016/j.jstrokecerebrovasdis.2017.06.009

15. Miller A, Pohlig RT, Reisman DS. Social and Physical Environmental Factors in Daily Stepping Activity in Those with Chronic Stroke. *Topics in stroke rehabilitation* (2021) 28:161-9. Epub 2020/08/11. doi: 10.1080/10749357.2020.1803571

16. Mudge S, Stott NS. Timed Walking Tests Correlate with Daily Step Activity in Persons with Stroke. *Arch Phys Med Rehabil* (2009) 90:296-301. doi: 10.1016/j.apmr.2008.07.025.

17. van de Port I, Punt M, Meijer JW. Walking Activity and Its Determinants in Free-Living Ambulatory People in a Chronic Phase after Stroke: A Cross-Sectional Study. *Disabil Rehabil* (2020) 42:636-41. doi: 10.1080/09638288.2018.1504327

18. Mahendran N, Kuys SS, Brauer SG. Which Impairments, Activity Limitations and Personal Factors at Hospital Discharge Predict Walking Activity across the First 6 Months Poststroke? *Disabil Rehabil* (2020) 42:763-9. doi: 10.1080/09638288.2018.1508513

19. Miller A, Pohlig RT, Reisman DS. Relationships among Environmental Variables, Physical Capacity, Balance Self-Efficacy, and Real-World Walking Activity Post-Stroke. *Neurorehabil Neural Repair* (2022) 36:535-44. doi:10.1177/15459683221115409

20. Danks KA, Pohlig RT, Roos M, Wright TR, Reisman DS. Relationship between Walking Capacity, Biopsychosocial Factors, Self-Efficacy, and Walking Activity in Persons Poststroke. *J Neurol Phys Ther* (2016) 40:232-8. doi: 10.1097/NPT.0000000000000143

21. French MA, Moore MF, Pohlig R, Reisman D. Self-Efficacy Mediates the Relationship between Balance/Walking Performance, Activity, and Participation after Stroke. *Top Stroke Rehabil* (2016) 23:77-83. doi: 10.1080/10749357.2015.1110306

22. Michael K, Macko RF. Ambulatory Activity Intensity Profiles, Fitness, and Fatigue in Chronic Stroke. *Top Stroke Rehabil* (2007) 14:5-12. doi:10.1310/tsr1402-5

23. Kanai M, Izawa KP, Kubo H, Nozoe M, Mase K, Shimada S. Association of Health Utility Score with Physical Activity Outcomes in Stroke Survivors. *Int J Environ Res Public Health* (2020) 18:251. doi:10.3390/ijerph18010251

24. Nayak P, Kumaran SD, Babu AS, Maiya AG, Solomon JM. Levels of Physical Activity and Quality of Life among Community-Dwelling Adults with Stroke in a Developing Country. *European Journal of Physiotherapy* (2019) 23:165-70. doi: 10.1080/21679169.2019.1663927

25. Ersöz Hüseyinsinoğlu B, Kuran Aslan G, Tarakci D, Razak Özdinçler A, Küçükoğlu H, Baybaş S. Physical Activity Level of Ambulatory Stroke Patients: Is It Related to Neuropsychological Factors? *Noropsikiyatri Arsivi* (2017) 54:155-61. doi: 10.5152/npa.2016.12760

26. Kossi O, Bonnechère B, Agbetou M, Somasse R, Hokpo A, Houehanou YCN, et al. Relationships between Cardiorespiratory Fitness, Physical Activity Practices, and Functional Outcomes One-Year Post-Stroke in Northern Benin: A Case–Control Study. *Top Stroke Rehabil* (2024) 31:104-15. doi: 10.1080/10749357.2023.2207286

27. Sekiguchi Y, Honda K, Izumi SI. Effect of Walking Adaptability on an Uneven Surface by a Stepping Pattern on Walking Activity after Stroke. *Front Hum Neurosci* (2022) 15. doi: 10.3389/fnhum.2021.762223.

28. Uçmak GS, Kilinç M. The Effects of Kinesiophobia, Fatigue, and Quality of Life on Physical Activity in Patients with Stroke. *Top Stroke Rehabil* (2024) 31:788-94. doi: 10.1080/10749357.2024.2333159

29. Paul L, Brewster S, Wyke S, Gill JMR, Alexander G, Dybus A, et al. Physical Activity Profiles and Sedentary Behaviour in People Following Stroke: A Cross-Sectional Study. *Disabil Rehabil* (2016) 38:362-7. doi: 10.3109/09638288.2015.1041615

30. Michael KM, Allen JK, Macko RF. Reduced Ambulatory Activity after Stroke: The Role of Balance, Gait, and Cardiovascular Fitness. *Arch Phys Med Rehabil* (2005) 86:1552-6. doi: 10.1016/j.apmr.2004.12.026
