# Supplementary material for: Contextual factors associated with walking performance after stroke: a systematic review and meta-analysis
Source: Front Neurol. 2025 Sep 24;16:1635024. doi: 10.3389/fneur.2025.1635024 (PMC12504098; doi:10.3389/fneur.2025.1635024)
Supplement: Supplementary file 8 [file Table_4.docx]

**Table S4.** Subgroup analysis of daily step counts in patients with stroke

| Subgroup | No. of studies | Pooled daily steps(95%CI) | | Q | Heterogeneity | | p-value across subgroups |
| --- | --- | --- | --- | --- | --- | --- | --- |
|  |  |  |  |  | I2（%） | P |  |
| Publication time |  |  | |  |  |  | 0.292 |
| 2005-2019 | 15 | 3977 (3135.431 - 4819.523) | | 433.56 | 0.968 | ＜0.001 |  |
| 2020-2024 | 12 | 4563 (3871.890 - 5253.761) | | 193.48 | 0.943 | ＜0.001 |  |
| Sample Size |  |  | |  |  |  | **0.01** |
| ≥50 | 11 | 4538 (3360.859 - 5714.966) | | 785.91 | 0.987 | ＜0.001 |  |
| 30-49 | 9 | 3215 (2642.331 - 3786.923) | | 68.65 | 0.883 | ＜0.001 |  |
| ＜30 | 7 | 5212 (3827.281 - 6596.278) | | 36.92 | 0.837 | ＜0.001 |  |
| Region |  |  |  |  |  |  | 0.745 |
| Europe | 4 | 4248 (3062.355 - 5433.143) | | 13.14 | 0.772 | ＜0.001 |  |
| Asia | 9 | 4680 (3565.475 - 5794.810) | | 263.99 | 0.970 | ＜0.001 |  |
| America | 12 | 3881 (2848.232 - 4914.365) | | 620.15 | 0.982 | ＜0.001 |  |
| Australasia | 2 | 4743 (2510.074 - 6976.063) | | 7.8 | 0.872 | 0.005 |  |
| Age |  |  |  |  |  |  | 0.978 |
| ＜65 | 16 | 4251 (3590.427 - 4912.523) | | 320.89 | 0.953 | ＜0.001 |  |
| ≥65 | 11 | 4232 (2999.001 - 5464.547) | | 451.29 | 0.978 | ＜0.001 |  |
| Time since stroke |  |  | |  |  |  | 0.215 |
| ＜3months | 2 | 3217 (1628.817 - 4805.504) | | 2.69 | 0.629 | 0.101 |  |
| 3-6months | 7 | 4729 (3971.831 - 5486.925) | | 29.82 | 0.799 | ＜0.001 |  |
| ＞6 months | 18 | 4196 (3445.838 - 4946.693) | | 737.81 | 0.977 | ＜0.001 |  |
| Measurement tool |  |  | |  |  |  | **0.003** |
| SAM/SWAB | 9 | 3967 (2727.622 - 5205.832) | | 285.09 | 0.972 | ＜0.001 |  |
| pedometer | 3 | 3331 (2156.636 - 4505.197) | | 13.38 | 0.851 | 0.001 |  |
| Fitbit | 8 | 5076 (4647.491 - 5504.303) | | 26.87 | 0.739 | ＜0.001 |  |
| accelerometer | 7 | 3733 (2839.297 - 4626.335) | | 81.46 | 0.926 | ＜0.001 |  |
