# Supplementary material for: Contextual factors associated with walking performance after stroke: a systematic review and meta-analysis
Source: Front Neurol. 2025 Sep 24;16:1635024. doi: 10.3389/fneur.2025.1635024 (PMC12504098; doi:10.3389/fneur.2025.1635024)
Supplement: Supplementary file 9 [file Table_5.docx]

**Table S5.** Meta-regression analysis of potential moderators of daily step counts in patients with stroke

| Variables | B | 95%CI | | R^2^ | *P* value |
| --- | --- | --- | --- | --- | --- |
|  |  | Lower | Upper |  |  |
| Publication time | 659.987 | -697.759 | 2017.733 | 0.29% | 0.326 |
| Sample Size | 0.953 | -9.485 | 11.391 | -4.70% | 0.852 |
| Region | -182.155 | -1018.012 | 653.703 | -4.58% | 0.657 |
| Age | -106.410 | -1506.266 | 1293.445 | -5.09% | 0.877 |
| Time since stroke | 48.894 | -1071.167 | 1168.955 | -5.07% | 0.929 |
| Measurement tool | 137.093 | -434.771 | 708.957 | -4.58% | 0.626 |
